# Multidimensional analysis and therapeutic development using patient iPSC–derived disease models of Wolfram syndrome

**DOI:** 10.1172/jci.insight.156549

**Published:** 2022-09-22

**Authors:** Rie Asada Kitamura, Kristina G. Maxwell, Wenjuan Ye, Kelly Kries, Cris M. Brown, Punn Augsornworawat, Yoel Hirsch, Martin M. Johansson, Tzvi Weiden, Joseph Ekstein, Joshua Cohen, Justin Klee, Kent Leslie, Anton Simeonov, Mark J. Henderson, Jeffrey R. Millman, Fumihiko Urano

**Affiliations:** 1 Department of Medicine, Division of Endocrinology, Metabolism, and Lipid Research, Washington University School of Medicine in St. Louis, St. Louis, Missouri, USA.; 2 Department of Biomedical Engineering, Washington University in St. Louis, St. Louis, Missouri, USA.; 3 National Center for Advancing Translational Sciences (NCATS), National Institutes of Health (NIH), Rockville, Maryland, USA.; 4 Dor Yeshorim, Committee for Prevention of Jewish Genetic Diseases, Brooklyn, New York, USA.; 5 Dor Yeshorim, Committee for Prevention of Jewish Genetic Diseases, Jerusalem, Israel.; 6 Amylyx Pharmaceuticals Inc., Cambridge, Massachusetts, USA.; 7 Department of Pathology and Immunology, Washington University School of Medicine in St. Louis, St. Louis, Missouri, USA.

**Keywords:** Endocrinology, Genetics, Beta cells, Diabetes, Genetic diseases

## Abstract

Wolfram syndrome is a rare genetic disorder largely caused by pathogenic variants in the *WFS1* gene and manifested by diabetes mellitus, optic nerve atrophy, and progressive neurodegeneration. Recent genetic and clinical findings have revealed Wolfram syndrome as a spectrum disorder. Therefore, a genotype-phenotype correlation analysis is needed for diagnosis and therapeutic development. Here, we focus on the *WFS1* c.1672C>T, p.R558C variant, which is highly prevalent in the Ashkenazi Jewish population. Clinical investigation indicated that patients carrying the homozygous *WFS1* c.1672C>T, p.R558C variant showed mild forms of Wolfram syndrome phenotypes. Expression of *WFS1* p.R558C was more stable compared with the other known recessive pathogenic variants associated with Wolfram syndrome. Human induced pluripotent stem cell–derived (iPSC-derived) islets (SC-islets) homozygous for *WFS1* c.1672C>T variant recapitulated genotype-related Wolfram syndrome phenotypes. Enhancing residual WFS1 function through a combination treatment of chemical chaperones mitigated detrimental effects caused by the *WFS1* c.1672C>T, p.R558C variant and increased insulin secretion in SC-islets. Thus, the *WFS1* c.1672C>T, p.R558C variant causes a mild form of Wolfram syndrome phenotypes, which can be remitted with a combination treatment of chemical chaperones. We demonstrate that our patient iPSC–derived disease model provides a valuable platform for further genotype-phenotype analysis and therapeutic development for Wolfram syndrome.

## Introduction

Wolfram syndrome is a rare, monogenic, life-threatening disease largely caused by pathogenic variants in the Wolfram syndrome (*WFS1*) gene, or in a small fraction of patients, pathogenic variants in the CDGSH iron sulfur domain protein 2 gene (1–3). There is currently no treatment to delay, halt, or reverse the progression of this disease. Wolfram syndrome is well characterized by juvenile-onset insulin-dependent diabetes, optic nerve atrophy, and progressive neurodegeneration (4, 5). Many patients also develop other symptoms, ranging from hearing loss and endocrine deficiencies to neurological and psychiatric conditions (4, 6). Accordingly, recent clinical and genetic findings have revealed that Wolfram syndrome is best characterized as a spectrum disorder (7). Of the approximately 200 *WFS1* variants associated with Wolfram syndrome, approximately 35% are missense, 25% are nonsense, 21% are frameshift, 13% are in-frame insertions or deletions, and 3% are splice-site variants (8, 9). Most of these variants are predicted to be inactivating, loss-of-function variants, but extensive molecular characterization of individual alleles is sparse. Hence, there is a great need for genotype-phenotype correlation data to guide diagnostic interpretation of *WFS1* variants.

*WFS1* encodes an ER transmembrane protein. The ER is a central cell organelle responsible for protein folding, Ca^2+^ storage, and lipid synthesis. It has been reported that WFS1 regulates Ca^2+^ homeostasis in the ER, which is crucial in the synthesis and secretion of neurotransmitters and hormones such as insulin (10, 11). WFS1 deficiency in the ER causes Ca^2+^ homeostasis disruption, leading to chronic ER stress followed by the unfolded protein response (UPR) (12, 13). WFS1 also negatively regulates activating transcription factor 6 (ATF6), a UPR molecule, inhibiting hyperactivation of ATF6 and consequent cell apoptosis (14). Furthermore, a recent study suggested that WFS1 affects mitochondrial function by transporting Ca^2+^ from the ER to the mitochondria via the mitochondria-associated ER membrane (15).

Several *Wfs1*-knockout rodents were developed as disease models of Wolfram syndrome, which generated insight into the etiology and provided opportunities to test therapeutic agents (11, 16–19). The models display progressive glucose intolerance due to impaired glucose-stimulated insulin secretion (GSIS) and increased pancreatic β cell death (16, 18–20). However, the onset of diabetes in these rodent models is delayed relative to the human phenotype, with further variation between each model based on rodent strain (16, 19). Also, to the best of our knowledge, there are no transgenic animals with phenotypes and variants corresponding to the pathogenic *WFS1* variants found in patients with Wolfram syndrome. As a result, these rodent models may not fully capture the spectrum of Wolfram phenotypes. By contrast, patient induced pluripotent stem cells (iPSCs) differentiated into disease-relevant cell types have been demonstrated as suitable models for genotype-phenotype correlation analysis (21, 22).

To date, significant efforts have been invested to develop novel Wolfram syndrome treatments (23). Compounds such as valproic acid and glucagon-like peptide-1 receptor agonists were identified as possible drug candidates based on preclinical studies in immortalized cell and rodent models (24–26). In addition, we recently conducted a phase Ib/IIa clinical trial of dantrolene sodium, an ER Ca^2+^ stabilizer, which demonstrated efficacy in a subset of patients with Wolfram syndrome (11, 27). However, additional therapeutic candidates for patients with Wolfram syndrome are still needed.

Here, we focus on the missense variant, *WFS1* c.1672C>T, p.R558C, which is enriched in the Ashkenazi Jewish population (allele frequency 1.4%) (28). We characterize this variant multidimensionally through clinical investigation, biochemical studies, and patient iPSC–derived disease models. Further, we demonstrate the potential efficacy of a combination treatment of chemical chaperones, sodium 4-phenylbutyrate (4-PBA) and tauroursodeoxycholic acid (TUDCA), as a potentially novel therapeutic approach for Wolfram syndrome.

## Results

### WFS1 c.1672C>T, p.R558C is enriched in the Ashkenazi Jewish population and causes a mild form of Wolfram phenotypes.

**Figure 1:**
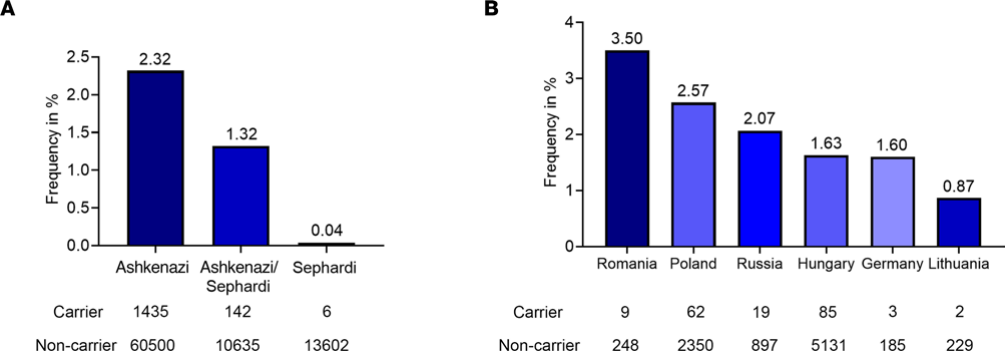
Carrier frequencies and clinical manifestation of *WFS1* c.1672C>T, p.R558C variant. (**A**) Carrier frequencies for *WFS1* c.1672C>T, p.R558C in patients of Ashkenazi, Ashkenazi/Sephardi, and Sephardi descent. (**B**) Carrier frequencies for *WFS1* c.1672C>T, p.R558C by country of origin.

To determine the carrier frequency for *WFS1* c.1672C>T, p.R558C variant in the Jewish population, we genotyped 87,093 patients from several Jewish populations. In the original data set, each patient was classified by self-identification as Ashkenazi, Sephardi, Ashkenazi/Sephardi, convert, and unknown. Samples from convert and unknown origin summed 773 and were excluded from analysis. The observed frequency of *WFS1* c.1672C>T, p.R558C carriers reached 2.32% (1:43) in Ashkenazi Jewish patients, 1.32% (1:76) for Ashkenazi/Sephardi patients, and 0.04% (1:2,268) in Sephardi Jewish patients (Figure 1A and Supplemental Table 1A; supplemental material available online with this article; https://doi.org/10.1172/jci.insight.156549DS1). To elucidate if *WFS1* c.1672C>T, p.R558C was present at higher rates in the Jewish population from various countries, we classified data based on self-reported ancestry of 4 grandparents. Patients who stated 2 or more countries of mixed origin were removed from analysis. In cases where South Africa was provided as the country of origin, the samples were redefined as Lithuanian, as South African Jews are primarily of Lithuanian origin (29). Patients who had Israel or United States listed in their ancestry were also removed because the Jewish people residing in these countries often have mixed Ashkenazi origins (30). Patients who did not provide any information on grandparental origin or stated unknown were removed. Patients with Ukrainian origin were merged into the Russian group. Patients with Belarus and Czechia origin were removed from analysis because they totaled fewer than 100 patients and a small sample group can produce spurious signals. In data classified by the country of origin, the frequencies occurred as follows: Romania 3.50% (1:29), Poland 2.57% (1:39), Russia 2.07% (1:48), Hungary 1.63% (1:61), Germany 1.60% (1:63), and Lithuania 0.87% (1:116) (Figure 1B and Supplemental Table 1B). Clinical investigation revealed that most patients carrying the homozygous *WFS1* c.1672C>T, p.R558C variant developed diabetes mellitus; however, the age at diagnosis was greater than that of typical Wolfram syndrome (approximately 6 years) (4) (Table 1). Only 4 patients were clinically diagnosed with optic nerve atrophy. Their optic nerve atrophy was mild, and no case was diagnosed as legally blind (Table 1). Additionally, no patient developed hearing loss or diabetes insipidus (Table 1). Together, *WFS1* c.1672C>T, p.R558C variant was enriched in the Ashkenazi Jewish population, especially those originated from Romania, and the variant led to mild or less severe phenotypes of Wolfram syndrome.

### The WFS1 p.R558C variant is degraded more than wild-type but less than WFS1 p.P885L variant.

**Figure 2:**
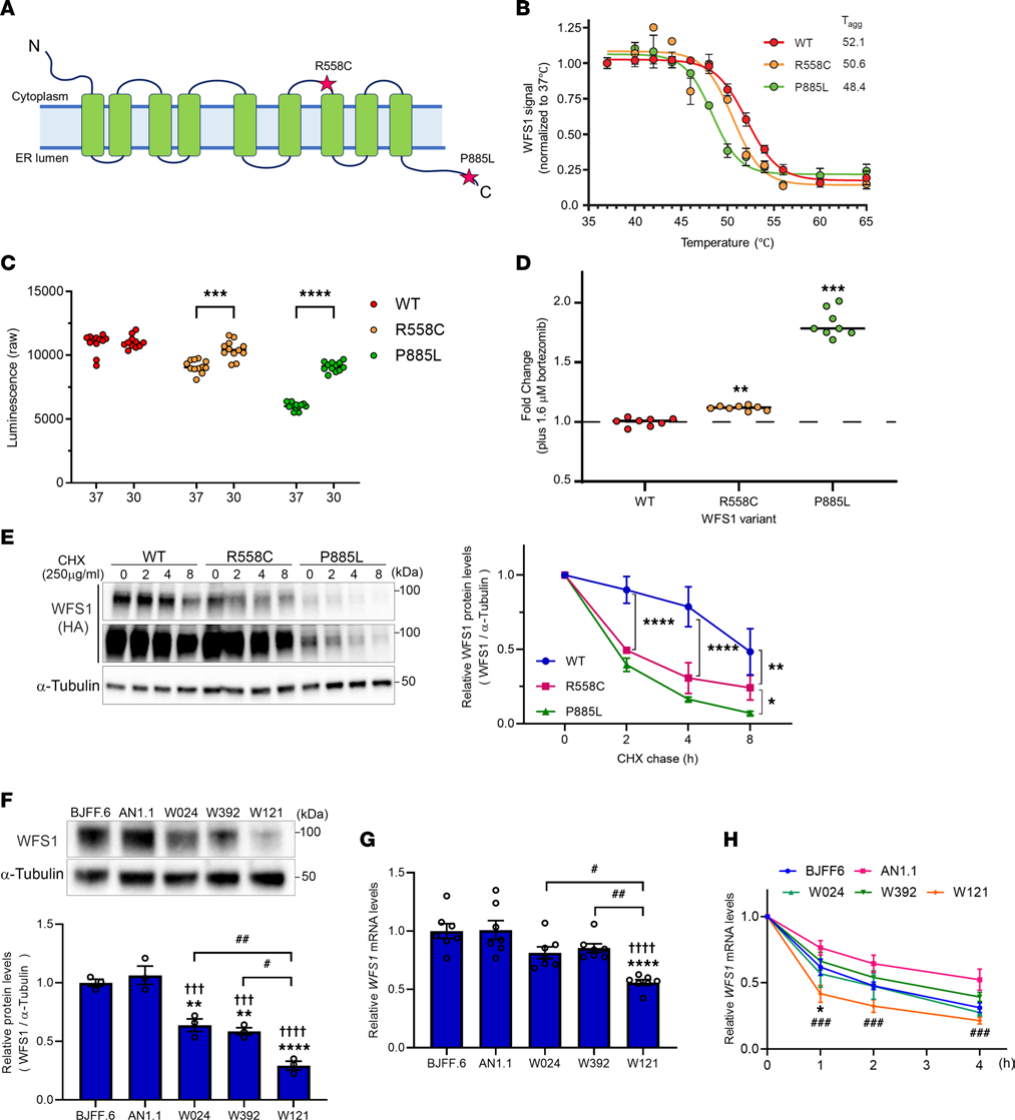
WFS1 p.R558C is more stable in the cell compared with p.P885L variant. (**A**) Diagram of WFS1 protein showing the location of 2 variants, R558C and P885L. (**B**) Thermal profiles of WFS1 variants (WT, R558C, and P885L) measured using SplitLuc-tagged reporters expressed in HEK293T cells (data from 3 independent experiments). (**C**) Luminescence intensities of WFS1 variants in cells incubated at 30°C and 37°C for 24 hours (*n* = 12, ***P < 0.001 and ****P < 0.0001 by unpaired *t* test). (**D**) Fold change of luminescence intensities of WFS1 variants treated with a proteasome inhibitor, bortezomib, for 24 hours (**P < 0.01 and ***P < 0.001 by unpaired *t* test compared with untreated). (**E**) (Left) Representative blotting image of WFS1 (HA) and α-Tubulin in CHX chase assay. Lower panel of WFS1 (HA) is long-exposure image. (Right) A quantification of relative WFS1 protein level normalized with α-Tubulin. (*n* =3, *P < 0.05, **P < 0.01, and ****P < 0.0001 by 2-way ANOVA.) (**F**) (Upper) Representative blotting image of WFS1 and α-Tubulin in iPSCs. (Lower) Quantification of relative WFS1 protein level normalized with α-Tubulin (*n* = 3, **P < 0.01 and ****P < 0.0001 by 1-way ANOVA compared with BJFF.6, ^†††^*P* < 0.001 and ^††††^*P* < 0.0001 by 1-way ANOVA compared with AN1.1, ^#^*P* < 0.05 and ^##^*P* < 0.01 by 1-way ANOVA). (**G**) Relative mRNA level of *WFS1* in iPSCs. (*n* = 7, ****P < 0.0001 by 1-way ANOVA compared with BJFF.6, ^††††^*P* < 0.0001 by 1-way ANOVA compared with AN1.1, ^#^*P* < 0.05 and ^##^*P* < 0.01 by 1-way ANOVA.) (**H**) Relative mRNA level of *WFS1* in ActD chase assay (*n* = 3, *P < 0.05 by 1-way ANOVA compared with BJFF.6, ^###^*P* < 0.001 by 1-way ANOVA compared with AN1.1).

Pathogenic *WFS1* variants are classified based on their effect on WFS1 expression: class A, depleted WFS1 protein or reduced, defective WFS1 protein, which leads to loss of function or incomplete function, or class B, expression of defective WFS1 protein leading to gain of function. Class A is further divided into 3 subclasses: class A1, WFS1 depletion due to *WFS1* mRNA degradation (nonsense-mediated decay, NMD); class A2, WFS1 depletion due to WFS1 protein degradation; or class A3, WFS1 depletion due to mRNA and protein degradation (31, 32) (Supplemental Figure 1). To determine the class specification of the *WFS1* c.1672C>T, p.R558C variant, we investigated the thermal stability of WFS1 p.R558C and p.P885L by appending a HiBiT-based tag (Promega) to detect the variant in cells (33). The p.R558C variant showed less thermal stability than wild-type WFS1, suggesting an altered folding state, but more stability compared with the known autosomal recessive variant p.P885L, which is pathogenic and is associated with a typical form of Wolfram syndrome (34, 35) (Figure 2, A and B). Both p.R558C and p.P885L expression could be rescued by incubating cells at reduced temperature, supporting a folding defect conferred by the variants (Figure 2C). Treatment with a proteasome inhibitor, bortezomib, increased WFS1 protein levels from both variants, and the fold change for p.P885L was higher than for p.R558C (Figure 2D), indicating that proteasomal degradation of p.R558C is less than p.P885L. To confirm this observation, we performed a cycloheximide (CHX) chase assay using HA-tagged WFS1 variants. After inhibiting translation of nascent protein by CHX treatment, the protein levels of p.R558C and p.P885L were rapidly decreased within 2 hours (Figure 2E). However, the rate of p.P885L decay was higher than p.R558C (Figure 2E). Also, the basal expression of p.P885L was lower before CHX treatment compared with wild-type and p.R558C, all consistent with more rapid degradation of p.P885L (Figure 2E).

Next, we examined if the *WFS1* variants endogenously expressed in cells would show similar posttranslational stabilities. We obtained peripheral blood mononuclear cells (PBMCs) from 3 patients carrying pathogenic variants in the *WFS1* gene (W024: c.1672C>T, c.1672C>T; W392: c.1672C>T, c.1672C>T; W121: c.1672C>T, c.2654C>T) and generated iPSCs (Table 2 and Supplemental Figure 2). Consistent with our clinical investigation, patients W024, W392, and W121 had mild phenotypes of Wolfram syndrome (Table 2). Western blot analysis revealed a reduction in WFS1 protein levels for W024, W392, and W121 compared with 2 control iPSC lines (BJFF.6 and AN1.1) (Figure 2F). Of the 3 patient lines, WFS1 protein level in W121 was less than W024 and W392 (Figure 2F). *WFS1* mRNA was not significantly decreased in W024 and W392 compared to control lines but was reduced for W121 (Figure 2G). We also performed the actinomycin D (ActD) chase assay to determine *WFS1* mRNA stabilities in each iPSC line. *WFS1* mRNA decay was higher than that in control line AN1.1, but similar to control line BJFF.6 (Figure 2H). On the other hand, corresponding to endogenous *WFS1* expression, *WFS1* mRNA in W121 was more unstable than both control lines (Figure 2H). Taken together, the *WFS1* c.1672C>T, p.R558C variant led to reduced expression of defective WFS1 protein, which was driven by posttranslation protein degradation, but not mRNA alterations, designating the variant as class A2.

### A combination treatment of 4-PBA and TUDCA ameliorates cellular function in neural progenitor cells with c.1672C>T, p.R558C variant.

**Figure 3:**
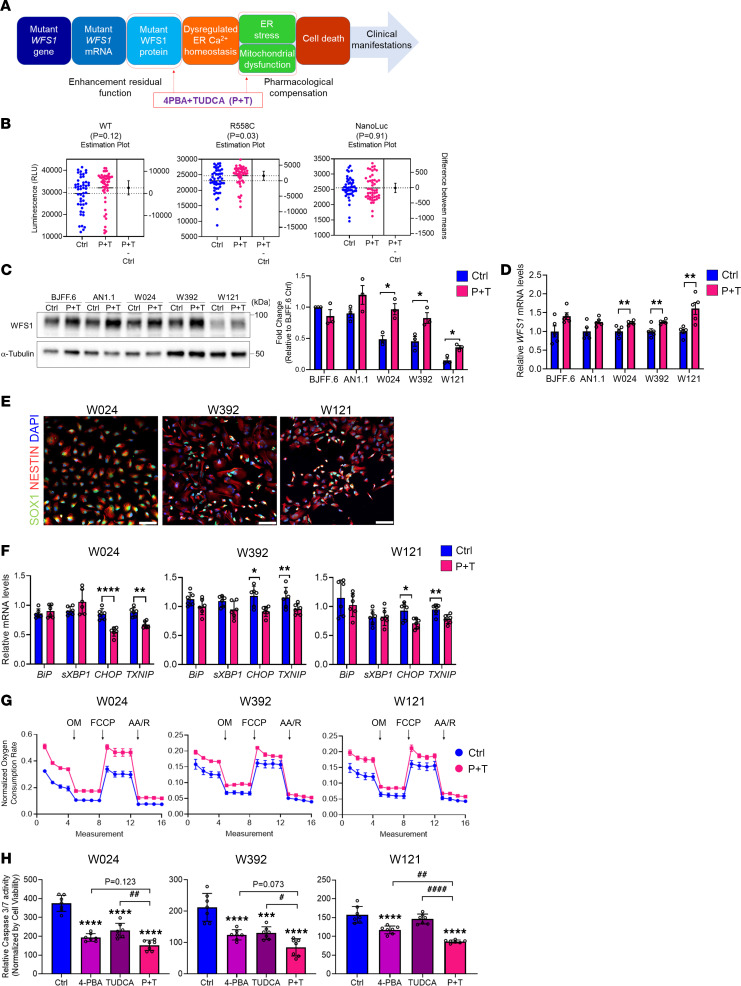
A combination treatment with 4-PBA and TUDCA mitigates detrimental effect of *WFS1* c.1672C>T, p.R558C variant. (**A**) A schematic of Wolfram syndrome etiology and the targets to modulate by a combination treatment of 4-PBA and TUDCA (P+T). (**B**) Expression of HiBiT-tagged WFS1 protein after treatment with 500 μM 4-PBA and 50 μM TUDCA (P+T) for 24 hours. NanoLuc levels, expressed from an identical plasmid backbone, were examined (*n* = 48, *P* value by unpaired *t* test). (**C**) (Left) Representative blotting images of WFS1 and α-Tubulin in iPSCs treated with or without P+T for 48 hours. (Right) Quantification of WFS1 protein levels normalized with α-Tubulin. (*n* = 3, *P < 0.05 by unpaired *t* test compared with Ctrl.) (**D**) Relative mRNA levels of *WFS1* in iPSCs treated with or without P+T for 48 hours (*n* = 5, **P < 0.01 by unpaired *t* test compared with Ctrl). (**E**) Representative immunofluorescence images of neural progenitor cell (NPC) markers in NPCs differentiated from patient-derived iPSCs. Scale bar: 100 μm. (**F**) Quantitative PCR analysis of ER stress–related genes in NPCs treated with or without P+T for 48 hours. (*n* = 6, *P < 0.05, **P < 0.01, and ****P < 0.0001 by unpaired *t* test compared with Ctrl.) (**G**) Mitochondrial respiration of NPCs treated with or without P+T for 48 hours represented as percentage of baseline oxygen consumption rate (OCR) measurements. Respiration was interrogated by measuring changes in relative OCR multiple times, every 8.5 minutes, after injection with oligomycin (OM), FCCP, and antimycin A (AA)/rotenone (R) (*n* = 3, W024: ***P < 0.001, W392: *P < 0.05, and W121: *P < 0.05 by unpaired *t* test compared with Ctrl AUC). (**H**) Caspase-3/7 activity normalized by cell viability in NPCs treated with or without either of 4-PBA, TUDCA, or P+T for 48 hours. (*n* = 7; ***P < 0.001 and ****P < 0.0001 by 1-way ANOVA compared with Ctrl; ^#^*P* < 0.05, ^##^*P* < 0.01, and ^####^*P* < 0.0001 by 1-way ANOVA.)

Wolfram syndrome is recognized as an ER disorder (6, 36, 37). Given that the ER and mitochondria interact both physiologically and functionally to maintain cellular homeostasis and determine cell fate under pathophysiological conditions, pathogenic *WFS1* variants cause not only ER dysfunction but also dysregulation of mitochondrial dynamics and appropriate function (38, 39). This evidence suggests that a combination drug modulating multiple targets simultaneously in the cell could be an effective treatment candidate for Wolfram syndrome. Chemical chaperones, such as 4-PBA and TUDCA, are well known to rescue or stabilize the native conformation of proteins by interacting with exposed hydrophobic segments of the unfolded protein (40). In addition to its chaperone activity, 4-PBA exhibits histone deacetylase–inhibitory (HDAC-inhibitory) activity, which transcriptionally induces the expression of molecular chaperones (41). TUDCA has been reported to reduce reactive oxygen species formation (42), prevent mitochondrial dysfunction (43), and inhibit apoptosis through the intrinsic (44) and extrinsic pathways (45). It has been shown that 4-PBA can improve insulin synthesis in iPSC-derived β cells from patients with typical Wolfram syndrome (46). We, therefore, hypothesized that a combination treatment of 4-PBA and TUDCA would have an additive effect on the restoration of reduced WFS1 expression and organelle dysfunction. Specifically, the combination treatment would be expected to affect pathogenic *WFS1* variants that target protein-level degradation, such as WFS1 p.R558C (Figure 3A).

We first tested if P+T stabilized WFS1 protein using the HiBiT-tagged reporters. The incubation with P+T significantly increased the steady-state levels of WFS1 p.R558C protein but not WT or a NanoLuc control expressed from an identical plasmid backbone (Figure 3B). Although the treatment slightly increased the steady-state levels of WFS1 p.P885L as well, it was not statistically significant (*P* = 0.0697, Supplemental Figure 3A). We also screened the NCATS Pharmaceutical Collection (~2,000 compounds), which includes approved drugs as well as 4-PBA, but not TUDCA, and found a small number of compounds that increased WFS1 p.R558C protein level, of which disulfiram was the top hit, but the magnitude of effect was similar to P+T (Supplemental Figure 3, B and C, and Supplemental Data 1). We next compared endogenous WFS1 protein levels in iPSCs treated with P+T. The P+T treatment significantly increased WFS1 protein levels in iPSCs derived from all 3 patient lines (Figure 3C). Of note, WFS1 protein levels in W024 and W392 were restored as great as control lines (Figure 3C). Additionally, mRNA level was increased by the P+T treatment (Figure 3D). We previously described organelle dysfunction preceding cell death in neural progenitor cells (NPCs) differentiated from iPSCs derived from patients with typical Wolfram syndrome (11). NPCs differentiated from the 3 patient lines and control line (AN1.1) expressed NPC markers NESTIN and SOX1 (Figure 3E and Supplemental Figure 4A). They showed a similar pattern of *WFS1* expression in iPSCs of these lines (Supplemental Figure 4B). Interestingly, the expression of ER stress marker genes, *BiP* and *spliced* *XBP1* (*sXBP1*), was not greatly changed among the lines, but *CHOP*, an ER stress–induced apoptosis gene, was significantly increased in each of the 3 patient lines (Supplemental Figure 4C). The other ER stress–induced apoptosis gene, *TXNIP*, was increased in W392 and W121 compared with AN1.1 (Supplemental Figure 4C).

Next, we examined if a combination treatment of 4-PBA and TUDCA would restore organelle functions in NPCs derived from the 3 patient iPSC lines. The expression of *BiP* and *sXBP1* was not affected by the P+T treatment, whereas *CHOP* and *TXNIP* were significantly decreased in each of the 3 patient lines (Figure 3F). We also measured the oxygen consumption rate (OCR) of NPCs to further assess mitochondrial function. Increased OCRs were observed throughout the assay in each of the 3 patient lines with the P+T treatment (Figure 3G). To investigate whether increased OCR was caused by increased mitochondrial number or improved mitochondrial function, we measured mitochondrial DNA contents and mitochondrial membrane potentials in the NPCs treated with or without P+T. Interestingly, mitochondrial DNA was increased in W024 and W392 NPCs by the treatment (Supplemental Figure 5A), whereas the mitochondrial membrane potentials were not affected (Supplemental Figure 5B). In W121 NPCs, both mitochondrial DNA and membrane potentials were increased by the treatment (Supplemental Figure 5, A and B). Besides these improvements, the P+T treatment inhibited apoptosis, as indicated by caspase-3/7 activity and cleaved caspase-3 protein levels, in each of the 3 patient lines (Figure 3H and Supplemental Figure 5C).

To define if a combination treatment of 4-PBA and TUDCA is valuable, we compared P+T efficacies with a single treatment with either 4-PBA or TUDCA. The P+T effect on endogenous WFS1 protein levels was the greatest in each of the 3 patient iPSC lines (Supplemental Figure 6A). Of note, WFS1 protein levels were significantly increased in W392 and W121 by the P+T treatment compared with a single treatment with each compound (Supplemental Figure 6A). The expression of ER stress–induced apoptosis genes was similar regardless of single or combination treatments in W024 and W392 NPCs, whereas only the P+T treatment significantly decreased ER stress–induced apoptosis gene expression in W121 (Supplemental Figure 6B). Mitochondrial DNA was not greatly changed by any single treatment in each of the 3 patient lines (Supplemental Figure 5A). Although we confirmed the increase of mitochondrial membrane potentials in W121 NPCs by the P+T treatment, it was not observed by a single treatment with each compound (Supplemental Figure 5B). Last, a single treatment with 4-PBA inhibited apoptosis in all 3 patient lines, and TUDCA also decreased apoptosis in W392 (Figure 3H and Supplemental Figure 5C). However, the magnitude of inhibition was the largest in the P+T treatment in each of 3 patient lines (Figure 3H and Supplemental Figure 5C). In addition, we confirmed the P+T treatment reduced caspase-3/7 activity in NPCs derived from patients with typical Wolfram syndrome (Supplemental Figure 7, A and B). In summary, a combination treatment of 4-PBA and TUDCA increased WFS1 expression and inhibited apoptosis by mitigating ER stress and mitochondrial dysfunction, which was more beneficial than a single treatment of either 4-PBA or TUDCA alone, though there were some variabilities among cell lines.

### A combination treatment of 4-PBA and TUDCA improves insulin secretion and survival in stem cell–derived β cells with WFS1 c.1672C>T, p.R558C variant.

**Figure 4:**
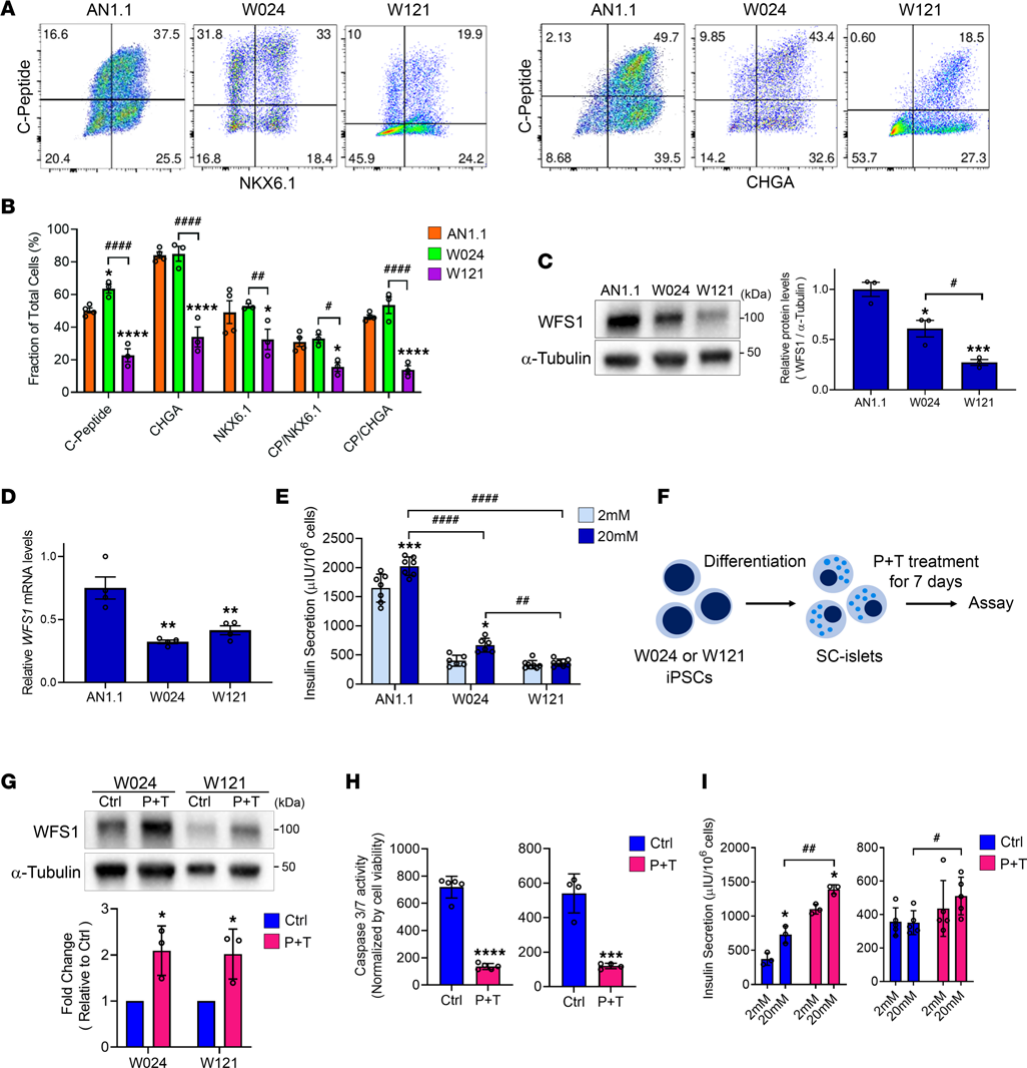
Insulin secretion is increased by a combination treatment of 4-PBA and TUDCA in SC-islets with *WFS1* c.1672C>T, p.R558C variant. (**A**) Representative flow cytometry dot plots and (**B**) quantified fraction of cells expressing or coexpressing pancreatic β cell or committed endocrine cell markers for AN1.1 (*n* = 4), W024 (*n* = 3), and W121 (*n* = 3) stage 6 SC-islets (*P < 0.05 and ****P < 0.0001 by 2-way ANOVA compared with AN1.1; ^#^*P* < 0.05, ^##^*P* < 0.01, and ^####^*P* < 0.0001 by 2-way ANOVA). (**C**) (Left) Representative blotting image of WFS1 and α-Tubulin in stage 6 SC-islets. (Right) Quantification of relative WFS1 protein level normalized with α-Tubulin (*n* = 3, *P < 0.05 and ***P < 0.001 by 1-way ANOVA compared with AN1.1; ^#^*P* < 0.05 by 1-way ANOVA). (**D**) Relative mRNA levels of *WFS1* in stage 6 SC-islets (*n* = 4, **P < 0.01 by 1-way ANOVA compared with AN1.1). (**E**) Static GSIS functional assessment of AN1.1 (*n* = 7), W024 (*n* = 6), and W121 (*n* = 8) stage 6 SC-islets (*P < 0.05 and ***P < 0.001 by 2-way ANOVA compared with 2 mM of each line; ^##^*P* < 0.01 and ^####^*P* < 0.0001 by 2-way ANOVA). (**F**) A schematic of P+T verification in SC-islets. (**G**) (Upper) Representative blotting images of WFS1 and α-Tubulin in stage 6 SC-islets treated with or without P+T for 7 days. (Lower) Quantification of WFS1 protein levels normalized with α-Tubulin. (*n* = 3, *P < 0.05 by unpaired *t* test compared with Ctrl.) (**H**) Caspase-3/7 activity normalized by cell viability in stage 6 SC-islets treated with or without P+T for 7 days (*n* = 3, ***P < 0.001 and ****P < 0.0001 by unpaired *t* test compared with Ctrl). (**I**) Static GSIS functional assessment of W024 (*n* = 5) and W121 (*n* = 4) treated with or without P+T for 7 days. (*P < 0.05 by by unpaired *t* test compared with 2 mM of each condition; ^#^*P* < 0.05 and ^##^*P* < 0.01 by 2-way unpaired *t* test.) CP, C-peptide.

The majority of patients with Wolfram syndrome develop diabetes mellitus due to the pathogenic *WFS1* variants causing detrimental effects in pancreatic β cells (13, 14, 20). To evaluate the impact of the *WFS1* c.1672C>T, p.R558C variant on β cells, we generated stem cell–derived pancreatic islets (SC-islets) from W024 and W121 iPSCs and AN1.1 iPSCs as a control. We previously developed a 6-stage differentiation strategy, incorporating cytoskeleton modulation, to produce SC-islets containing hormone-secreting endocrine cell types, including insulin-positive stem cell–derived β (SC-β), glucagon-positive stem cell–derived α, and somatostatin-positive stem cell–derived δ cells (47, 48) (Supplemental Figure 8A). The W024 and W121 stage 6 SC-islets produced C-peptide^+^ cells coexpressing β cell differentiation marker (NKX6.1) and committed endocrine cell marker (chromogranin A, CHGA). The β cell population was similar between W024 and control SC-islets but reduced in the W121 line (Figure 4, A and B). WFS1 protein was expressed in SC-islets derived from all 3 lines, with greater expression detected in control SC-islets (Figure 4C). Of note, WFS1 protein level was significantly higher in W024 SC-islets when compared with W121 (Figure 4C). However, both patient-derived SC-islets (W024 and W121) showed a significant reduction of *WFS1* mRNA levels (Figure 4D), which was not observed in W024 iPSCs and NPCs (Figure 2G and Supplemental Figure 4B). We previously demonstrated the robust increase of *WFS1* expression during the SC-islet differentiation from stage 5 to stage 6 (49), suggesting that *WFS1* expression in SC-islets could be much higher than iPSCs and NPCs. Previous studies showed WFS1 deficiency causes mild dilation of the ER in β cells (19, 50, 51). Electron microscopic analyses displayed well-formed ER structures in AN1.1 SC-islets (Supplemental Figure 9). On the other hand, ERs in W024 and W121 SC-islets were distorted, fragmented, and dilated (Supplemental Figure 9). We tested the functional capacity of the SC-islets in response to high glucose (20 mM) using the GSIS assay. Throughout GSIS, W024 and W121 SC-islets secreted less insulin compared with control SC-islets. W024 SC-islets were able to increase their insulin secretion in response to the glucose stimulus, whereas W121 SC-islets were not capable of a glucose-stimulated response (Figure 4E). These data suggest the *WFS1* c.1672C>T, p.R558C variant has a milder effect on β cell insulin secretion than the *WFS1* c.2654C>T, p.P885L variant.

Next, we tested if the P+T treatment is effective in ameliorating W024 and W121 SC-islet dysfunction (Figure 4F). WFS1 protein expression was restored in the treated SC-islets, as observed in both the W024 and W121 iPSCs (Figure 4G). We observed the dilated ER was remitted in some W024 and W121 SC-islet cells treated with P+T (Supplemental Figure 9). In addition, the P+T treatment greatly inhibited cell death in the W024 and W121 SC-islets (Figure 4H). As expected with greater WFS1 protein, the insulin secretion of W024 and W121 SC-islets in low and high glucose was increased by the P+T treatment (Figure 4I). However, insulin content was increased only in W121 SC-islets by the P+T treatment (Supplemental Figure 8B). The proinsulin/insulin ratio was not changed in either W024 or W121 SC-islets by the treatment (Supplemental Figure 8C), suggesting P+T does not alter insulin processing. We also measured OCRs of W024 and W121 treated with P+T. Although W024 showed a trend of slight increase in oxygen consumption over the assay by P+T treatment, neither W024 nor W121 showed significant improvement by the treatment (Supplemental Figure 8D). In summary, P+T treatment restored WFS1 expression and increased insulin secretion capabilities of W024 and W121 SC-islets.

### Cellular stress is mitigated by a combination treatment of 4-PBA and TUDCA in SC-islets with WFS1 c.1672C>T, p.R558C variant.

**Figure 5:**
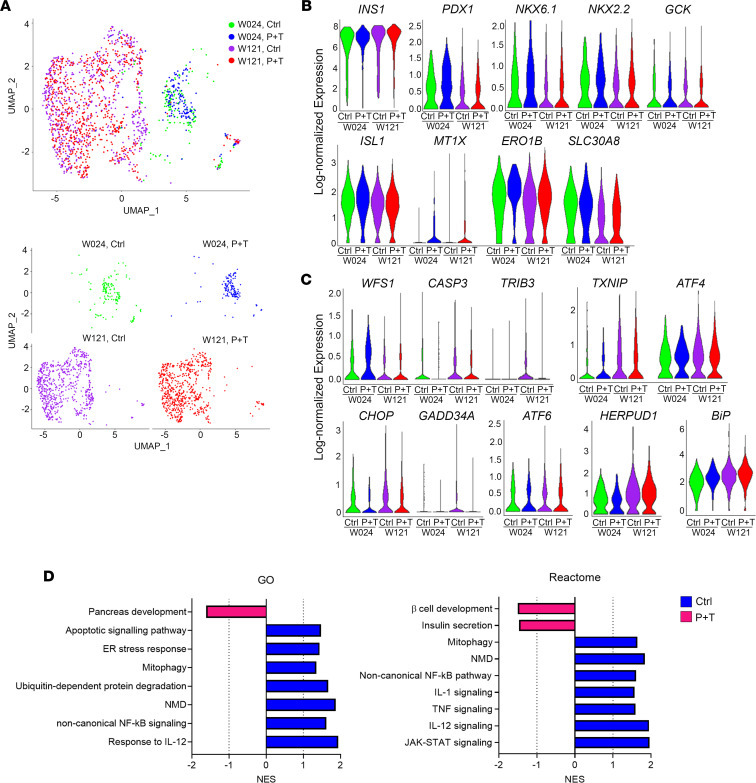
Single-cell transcriptional evaluation of a combination treatment with 4-PBA and TUDCA on SC-β cells. (**A**) Uniform manifold approximation and projection (UMAP) plot from unsupervised clustering of combined transcriptional data from scRNA-Seq of W024, Ctrl (green); W024, P+T (blue); W121, Ctrl (purple); and W121, P+T (red) SC-β cell populations. Lower plots are UMAP plots split by experimental conditions. (**B**) Violin plots detailing log-normalized gene expression of β cell genes in the same populations as **A**. Log fold change and *P* values for violin plots are available in Supplemental Table 2. (**C**) Violin plots detailing log-normalized gene expression of ER stress and apoptotic genes in the same populations as **A**. Log fold change and *P* values for violin plots are available in Supplemental Table 3. (**D**) Gene Ontology (GO) and Reactome GSEA, quantified by the normalized enrichment score (NES), for pathways upregulated in the combined population of W024 and W121 SC-β cells treated with (pink) or without (blue) P+T. NES values, *P* values, FDR *q* values, and gene set lists are available in Supplemental Table 4.

We performed multiplexed single-cell RNA sequencing (scRNA-Seq) using the 10x Genomics platform to investigate genotype-phenotype correlations and the efficacy of the P+T combination treatment on SC-β cells more precisely. We utilized cell hashing, which applies oligo-tagged antibodies to the cell surface proteins of individual samples, thus allowing detection of individual samples within a pooled cell population (52). We sequenced 4 biological replicates per cell line from independent differentiations, treated with or without P+T for 7 days. In total, we sequenced 16 samples with 8 samples in each pooled population, which were submitted separately based on the cell line. In total, we sequenced 13,951 stage 6 SC-islet cells differentiated from W024 and W121 iPSCs to study the effects of P+T treatment (W024: 2,619 cells; W024, P+T: 3,158 cells; W121: 3,625 cells; and W121, P+T: 4,549 cells; 4 biological replicates for each sample). The scRNA-Seq data were analyzed using dimensionality reduction and unsupervised clustering to classify individual cells into cell populations based on similarities in their transcriptome profiles. The cell types were identified by aligning the top upregulated genes in each cell cluster population with published pancreatic transcriptome data (53, 54). After identifying the β cell population in each sample, we combined the 2,329 SC-β cells from the 4 experimental conditions (W024: 377 cells; W024, P+T: 220 cells; W121: 749 cells; and W121, P+T: 680 cells) and performed principal component analysis and unsupervised clustering. The β cells clustered together based on genetic background, regardless of combination treatment (Figure 5A), suggesting the β cell transcriptional profile was not greatly changed in response to P+T.

Next, we evaluated the key β cell genes and ER stress markers. *WFS1* pathogenic variants cause ER stress, resulting in altered expression of β cell genes in SC-β cells (49). Expression of the insulin gene (*INS*), crucial transcription factors for β cell differentiation (*ISL1*, *NKX6.1*, *NKX2.2*, and *PDX1*), β cell maturation genes (*MT1X* and *ERO1B*), and a β cell function gene (*GCK*) was similar between untreated W024 and W121 SC-β cells (Figure 5B and Supplemental Table 2). Interestingly, the expression of *SLC30A8*, an alternate β cell maturation gene, was significantly higher in W024 SC-β cells compared with W121 (Figure 5B and Supplemental Table 2). The P+T treatment did not change the gene expression for many genes in W024 SC-β cells (Figure 5B and Supplemental Table 2). On the other hand, *MT1X* and *ERO1B* were highly expressed in W121 SC-β cells treated with P+T compared with untreated (Figure 5B and Supplemental Table 2). Unlike WFS1 protein levels in SC-islets, *WFS1* transcription within the SC-β cell population was similar between both W024 and W121 SC-β cells (Figure 4C, Figure 5C, and Supplemental Table 3). Some ER stress markers (*TXNIP* and *BiP*) were highly expressed in W121 SC-β cells compared with W024 (Figure 5C and Supplemental Table 3), whereas other ER stress markers (*ATF6*, *ATF4*, *CHOP*, *GADD34A*, *TRIB3*, and *HERPUD1*) and apoptotic (*CASP*) genes were not statistically different between both W024 and W121 SC-β cells (Figure 5C and Supplemental Table 3). The expression of *WFS1*, ER stress markers, and apoptotic genes was not statistically altered by the P+T treatment in either W024 or W121 SC-β cells (Figure 5C and Supplemental Table 3).

We performed gene set enrichment analysis (GSEA) on the SC-β cells. Gene sets pertaining to NMD, ubiquitination-mediated protein degradation, and oxidative stress were enriched in the untreated SC-β cells compared with the P+T-treated SC-β cells (Figure 5D and Supplemental Table 4). Interestingly, we found the inflammation and the selective mitochondrial autophagy (mitophagy) pathways were also enriched in the untreated SC-β cell population (Figure 5D, Supplemental Figure 10, and Supplemental Table 4). Although we did not observe major changes in the expression of ER stress markers and apoptotic genes in W024 and W121 SC-β cells treated with P+T, gene sets pertaining to apoptosis and ER stress were enriched in the untreated SC-β cells (Figure 5D, Supplemental Figure 10, and Supplemental Table 4). Of note, gene sets related to insulin secretion and β cell development were enriched in the P+T-treated SC-β cell population (Figure 5D, Supplemental Figure 10, and Supplemental Table 4). Additionally, gene sets related to regulation of cytosolic K^+^ and Ca^2+^ levels were increased, and these levels play an important role in β cell differentiation and function (55–57) (Supplemental Figure 10 and Supplemental Table 4). Collectively, P+T treatment mitigated cellular stress increased by pathogenic *WFS1* variants without changing β cell identity, which resulted in increased β cell and insulin secretion in W024 and W121 SC-β cells.

### A combination treatment of 4-PBA and TUDCA delays the diabetic phenotype progression in Wfs1-deficient mice.

**Figure 6:**
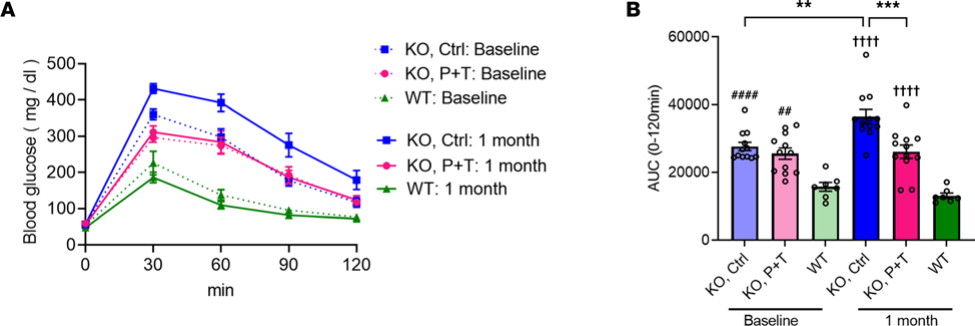
In vivo verification of a combination treatment with chemical chaperones. (**A**) Intraperitoneal glucose tolerance test (IP-GTT) with WT or *Wfs1*-KO mice at baseline and 1 month after feeding with either control chow or food containing 4-PBA: 0.338% and TUDCA: 0.225% (P+T chow). (**B**) AUCs of the IP-GTT (KO, Ctrl: *n* = 12; KO, P+T: *n* = 12; WT: *n* = 7; **P < 0.01 and ***P < 0.001 by 1-way ANOVA; ^##^*P* < 0.01 and ^####^*P* < 0.0001 by 1-way ANOVA compared with WT: Baseline; ^††††^*P* < 0.0001 by 1-way ANOVA compared with WT: 1 month).

Finally, we verified the efficacy of our combination treatment with chemical chaperones with an in vivo study. The field lacks a c.1672C>T, p.R558C variant *WFS1* mutation mouse model. Therefore, we employed 129S6 whole-body *Wfs1*-knockout (*Wfs1*-KO) mice. This mouse model develops progressive glucose intolerance during adolescence, hence a mouse model of Wolfram syndrome (20). We showed *Wfs1*-KO mice developed glucose intolerance at 5–6 weeks old (Figure 6, A and B). In addition, *Wfs1*-KO mice did not show glucose-stimulated increase of the serum insulin level, which was lower than that of WT (Supplemental Figure 11A). We treated the mice at 5–6 weeks old with P+T chow (4-PBA: 0.338% and TUDCA: 0.225%) for 1 month. Both groups of *Wfs1*-KO mice consumed similar amounts of chow (Supplemental Figure 11B). After feeding for 1 month, *Wfs1*-KO mice fed with control chow developed more severe glucose intolerance (Figure 6, A and B). Conversely, an IP-GTT blood glucose curve was similar to the baseline outcome in *Wfs1*-KO mice fed with P+T chow (Figure 6, A and B), indicating that P+T chow delayed the progression of the diabetic phenotype. Body weight and insulin sensitivity were not greatly changed by the P+T chow (Supplemental Figure 11, C–E). The basal level of serum insulin (0 minutes) was higher in *Wfs1*-KO mice fed with P+T chow as compared with control chow (Supplemental Figure 11F). Compared with baseline, serum insulin level at 30 minutes following glucose injection was decreased in *Wfs1*-KO mice fed with control chow, whereas it was similar in the mice fed with P+T chow (Supplemental Figure 11G). Collectively, we observed delays in the Wolfram diabetic phenotype in *Wfs1*-KO mice when using the P+T treatment. Therefore, we expect the combination treatment to be efficacious against diabetic Wolfram phenotypes caused by the *WFS1* c.1672C>T, p.R558C variant in vivo.

## Discussion

In this study, we characterize a likely unique pathogenic *WFS1* c.1672C>T, p.R558C variant, which is associated with a mild form of Wolfram syndrome and highly prevalent in the Ashkenazi Jewish population. Molecular investigation revealed WFS1 p.R558C had a greater posttranslational stability in cells compared with another pathogenic variant, *WFS1* c.2654C>T, p.P885L. Based on the molecular characterization of WFS1 p.R558C, we hypothesized a combination treatment of 2 chemical chaperones, 4-PBA and TUDCA, could provide a treatment for Wolfram syndrome. We demonstrated that a combination treatment of 4-PBA and TUDCA increased WFS1 expression within the cell, ameliorating organelle dysfunction and the associated apoptosis. We also identified that patient SC-islets demonstrated genotype-phenotype relationships that correlated with clinical observations, and a combination treatment with 4-PBA and TUDCA improved insulin secretion in this cellular model of Wolfram syndrome.

Wolfram syndrome is a very rare genetic disorder. In the United Kingdom, the prevalence is 1:770,000, and the carrier frequency of pathogenic *WFS1* variants is 1:354 (4). The prevalence in the North American population is estimated to be 1:100,000 (58). We observed greater carrier frequency of the *WFS1* c.1672 C>T, p.R558C variant in the Ashkenazi Jewish population with a frequency of 1:43 as compared with 1:76 in the Ashkenazi/Sephardi Jewish population and 1:2,268 in the Sephardi population. Although present in the Sephardi population, as shown in 6 carriers out of 13,608 Sephardi patients tested, we suspect that the low frequency of *WFS1* c.1672 C>T, p.R558C variant might be explained by historical admixture with Ashkenazim. Forgotten or suppressed admixture of Gentile and Jewish people was previously exhibited in a study focusing on genome-wide Jewish genetic signature (59).

WFS1 is subjected to a ubiquitin-proteasomal degradation by SMAD specific E3 ubiquitin protein ligase 1 (Smurf1), a HECT-type ligase, which recognizes the degron within the C-terminal luminal region of WFS1 (aa 671–700) (60). Missense mutations in the degron or truncating mutations lacking the degron are resistant to Smurf1-mediated degradation (60). Other *WFS1* variants that retain the functional degron can lead to complete depletion or degradation of the WFS1 protein by NMD or proteasomal degradation (31, 32). Our study demonstrated WFS1 p.R558C protein is also subjected to proteasomal degradation. However, the degradation rate was lower than the WFS1 p.P885L variant. Additionally, WFS1 p.R558C protein was detected while conducting in vivo and in vitro experiments, indicating that a portion of the WFS1 p.R558C protein escapes proteasomal degradation. The remaining WFS1 p.R558C protein may compensate for the loss of wild-type WFS1 function. This hypothesis is consistent with the observation that clinically, patients carrying the homozygous *WFS1* c.1672 C>T, p.R558C variant have mild or less severe phenotypes of Wolfram syndrome. Our findings strongly suggest that alternate degron-retaining pathogenic *WFS1* variants require further protein expression characterization to determine the genotype-phenotype correlation.

Most of the studies conducting experimental genotype-phenotype correlation analysis employ overexpression of mutant WFS1 proteins in transfected cell lines such as HEK293T, HeLa, and COS7 (60, 61). This system is incapable of evaluating detrimental effects of pathogenic *WFS1* variants on physiological functions in disease-relevant cells. In pursuit of more reliable models and achieving a deeper understanding of Wolfram phenotypes, we have studied patient-derived, disease-relevant cells. SC-islets, containing SC-β cells, differentiated from patient iPSCs with homozygous *WFS1* c.1672C>T, p.R558C variant (W024), displayed insufficient β cell function and similar differentiation efficiency compared to control SC-islets. W024 SC-islet phenotypes were milder than W121 SC-islets differentiated from the heterozygous *WFS1* c.1672C>T, c.2654C>T variant iPSC line, indicating that our SC-islets capture Wolfram phenotypes based on pathogenic *WFS1* variants and could model genotype-phenotype correlation analysis and drug discovery in vitro. The diabetes stem cell field has recently leveraged this technology to correct the WFS1 pathogenic variant using CRISPR/Cas9 gene-edited SC-islets from a patient with Wolfram syndrome to reverse preexisting diabetes in a mouse (49). Stem cell technology and genetic engineering have also been used for correcting and studying other pathogenic variants (62).

We demonstrated that a combination treatment of 4-PBA and TUDCA was efficacious against the *WFS1* c.1672C>T variant. In our screening, the disulfiram molecule stabilized the WFS1 protein. The stabilization is likely due to the inhibitory function of disulfiram on NPL4, an adaptor of p97/VCP segregase, which is essential for ER-associated degradation (ERAD) (63, 64). ERAD is a crucial system aiming to mitigate ER stress. Indeed, some studies showed that disulfiram induces ER stress followed by apoptosis (65, 66). On the other hand, 4-PBA and TUDCA stabilize proteins by facilitating protein folding thereby mitigating ER stress. Numerous studies have demonstrated that 4-PBA and TUDCA are promising for the treatment of diabetes mellitus and other ER stress–related neurodegenerative diseases (67–70). However, most chaperone treatment studies used an individual chaperone and a single-dose treatment. A combination treatment with these compounds has rarely been attempted. Combining several compounds is a common cancer treatment strategy, aiming to yield additive or synergistic efficacy (71). Recently, a study reported that a combination treatment of 4-PBA and TUDCA resulted in slower functional decline of patients with amyotrophic lateral sclerosis, an ER stress–related disease (72). In our study, 4-PBA and TUDCA increased WFS1 protein as well as mRNA levels in the patient-derived iPSCs. We predict this increase of *WFS1* mRNA level is induced by 4-PBA activity as an HDAC inhibitor, which selectively promotes gene transcription (41, 73). Alternatively, *WFS1* and ER stress marker expression in SC-β cells was not statistically significantly altered by the combination treatment with 4-PBA and TUDCA in our scRNA-Seq analysis. This could be due to variability in the scRNA-Seq data set. Genes not in a consistent steady-state manner cause variable detection, which is commonly observed in ER stress–inducible genes (74, 75). Additionally, current scRNA-Seq technology detects only approximately 10% of the cellular mRNA molecules, resulting in difficulties in detecting genes with small expression within a single cell (76). Therefore, the scRNA-Seq data set may be limited in the number of gene transcript copies detected for ER stress markers and *WFS1*, thus underestimating the impact of 4-PBA and TUDCA on ER stress in the SC-β cells.

GSEA revealed a combination treatment of 4-PBA and TUDCA downregulated mitophagy, ER stress, and apoptosis. Mitophagy mediates clearance of damaged mitochondria in the cells (77). Pathogenic *WFS1* variants have been reported to cause mitochondrial dysfunction (38, 39), and we showed a combination treatment of 4-PBA and TUDCA restored mitochondrial function in NPCs, implying that mitophagy could be reduced by preventing mitochondrial dysfunction. Additionally, in GSEA, several inflammatory pathways were reduced by a combination treatment of 4-PBA and TUDCA. We and others recently reported elevated expression and serum levels of inflammatory cytokines in patients with Wolfram syndrome (27, 78). ER stress and mitochondrial dysfunction have been well known to cause inflammation (79–82). It is possible that the several inflammatory pathways were downregulated as a consequence of reduced ER stress and restored organelle function by a combination treatment of 4-PBA and TUDCA. In summary, a combination treatment of 4-PBA and TUDCA mitigated various cellular stresses the *WFS1* c.1672C>T, p.R558C variant caused, resulting in improved insulin secretion.

We previously reported on the restoration of Wolfram phenotypes by correcting pathogenic *WFS1* variants with CRISPR/Cas9 in SC-islets derived from patients with typical Wolfram syndrome (49). When comparing the functional assessments and scRNA-Seq analyses, a combination treatment of 4-PBA and TUDCA had a milder effect compared with CRISPR/Cas9 gene correction. This is expected because CRISPR/Cas9 correction directly intervenes on the cause of Wolfram syndrome. However, the combination treatment of 4-PBA and TUDCA as a therapeutic is advantageous for ease of administration. Furthermore, prolonged 4-PBA and TUDCA administration could delay additional symptom onset, including hearing loss and neurodegeneration, which typically manifests at later stages of the disease. Therefore, verifying efficacy of the combination treatment on other relevant cell types and pathogenic *WFS1* variants is warranted.

This study harnesses multiple iPSC-derived in vitro disease models and demonstrates efficacy of a combination treatment of 4-PBA and TUDCA. The benefits of this treatment may apply to other genetic ER stress–related diseases, such as Wolcott-Rallison syndrome. Moreover, our differentiation technology for in vitro disease models could be leveraged as a drug screening tool to discover new therapies or pave the way to personalized medicine strategies for patients with Wolfram syndrome.

## Methods

### iPSC lines.

To generate iPSCs, we obtained PBMCs from patients with Wolfram syndrome. The iPSC lines were generated by the Genome Engineering and iPSC Center (GEiC) at Washington University in St. Louis with Sendai viral reprogramming. Control iPSC lines BJFF.6 and AN1.1 were obtained from the GEiC at Washington University in St. Louis. HEK293 cells were obtained from ATCC. Validation of pluripotency was performed using Pluripotent Stem Cell 4-Marker Immunocytochemistry Kit (Thermo Fisher Scientific; A24881).

### Chemical chaperones.

We obtained 4-PBA and TUDCA from Amylyx Pharmaceuticals Inc. We dissolved 4-PBA and TUDCA in PBS and used them at final concentrations of 500 μM and 50 μM, respectively. Control conditions were treated with only PBS. The treatment time is described in the figure legends. Stock reagents were kept at 4°C and made fresh every 2 weeks. For animal experiments, we ordered Envigo custom-made chow containing 4-PBA and TUDCA (4-PBA: 34 mg/kg and TUDCA: 23 mg/kg in Teklad global 18% protein rodent diet). The animals ate approximately 4 g of diet per day, with a goal of 6 g 4-PBA/kg body weight/d and 4 g TUDCA/kg body weight/d. The same base diet without chemical chaperones was used as the control chow.

### NPC differentiation.

Undifferentiated stem cells were plated down on tissue culture plastic in mTeSR1 (StemCell Technologies; 05850) and cultured in a humidified 5% CO_2_ and 37°C tissue culture incubator. NPC differentiation was performed as described previously (83, 84). Briefly, 4 × 10^4^ to 6 × 10^4^ cells per well were seeded onto V-bottom, 96-well plates (Corning) in NPC differentiation medium suppled with 10 μM Y27632 (Tocris; 1254) to generate embryonic bodies (EBs) (day 0). On day 4, EBs were plated on 6 cm dishes coated with poly-l-ornithine solution (20 μg/mL, MilliporeSigma) and laminin (1 μg/mL, Thermo Fisher Scientific) in NPC differentiation medium. On day 12, neural rosettes were detached from the dish using STEMdiff Neural Rosette Selection Reagent (StemCell Technologies; 05832), and dissociated cells (NPCs) were plated on 6 cm dishes coated with Matrigel and laminin (5 μg/mL). NPC differentiation medium consists of Neurobasal-A (Life Technologies; 10888022), 1× B27 supplement without vitamin A (Life Technologies; 12587), 1% nonessential amino acids (Life Technologies; 11140050), 0.1% 2-mercaptoethanol (Life Technologies; 21985-023), 1% PenStrep (Life Technologies; 15140122), 1% Glutamax (Life Technologies 35050061), 10 μM SB-431542 (Tocris; 1614), and 100 nM LDN-193189 (Tocris; 6053). After the differentiation, medium was changed every other day in the whole differentiation process. NPCs were maintained in STEMdiff Neural Progenitor Medium (StemCell Technologies; 05833). Passage was performed with Accutase (MilliporeSigma; A6964) and medium was changed every day.

### SC-islet differentiation.

SC-islet differentiation was performed similarly to as we have previously reported (47, 48). Undifferentiated stem cells were plated down on tissue culture plastic in mTeSR1 and cultured in a humidified 5% CO_2_, 37°C tissue culture incubator. Stem cell passaging occurred every 3–4 days, with TrypLE (Life Technologies; 12-604-039) used for single-cell dispersion and Vi-Cell XR (Beckman Coulter) for counting. Undifferentiated stem cells were seeded at 0.52 × 10^6^ cells/cm^2^ in mTeSR1 + 10 μM Y27632 (Abcam; ab120129) on Matrigel-coated (Corning; 356230) plates. On stage 6 day 7, cells were single-cell dispersed with TrypLE and seeded in a 6-well plate at 5 × 10^6^ cells per well with 4 mL of stage 6 enriched serum-free medium per well. Cells continued culturing on an orbi-shaker (Benchmark) set at 100 rpm in a humidified tissue culture incubator at 5% CO_2_ and 37°C. Supplemental Table 7 contains subsequent feeding schedule, media formulations, and differentiation factors. Assays were carried out between stage 6 day 9 and 20.

### Data availability.

RNA-Seq data were deposited in the NCBI’s Gene Expression Omnibus database (GEO accession GSE212256).

### Statistics.

Statistical analysis was performed by 1-tailed unpaired and paired *t* tests and 1- and 2-way ANOVA with Tukey’s or Dunnett’s tests. Statistical tests are specified in figure legends. *P* < 0.05 was considered statistically significant. Data are shown as means ± SEM unless otherwise noted.

### Study approval.

For human study, patients and their parents or legal guardians, as appropriate, provided written informed consent before participating in this study, which was approved by the Human Research Protection Office at Washington University School of Medicine in St. Louis (IRB ID 201107067). Animal experimentation was performed according to procedures approved by the Institutional Animal Care and Use Committee at the Washington University School of Medicine in St. Louis (20-0334).

Further methods details are available in the Supplemental Methods.

## Author contributions

RAK, KGM, JRM, and FU conceived the experimental design. YH, MMJ, TW, JE, and FU conducted clinical investigation. WY, MJH, and AS performed WFS1 SplitLuc assays and screening. KGM differentiated β cells and performed functional assays. KGM and PA performed sequencing analysis. RAK and CMB performed animal experiments. RAK differentiated NPCs and RAK, CMB, and KK performed the other in vitro experiments. JC, JK, and KL examined data on 4-PBA+TUDCA. JRM and FU supervised the data. RAK, KGM, and FU wrote the manuscript. All authors edited and reviewed the manuscript.

## Supplementary Material

Supplemental data

Supplemental data set 1

## Figures and Tables

**Table 2:**
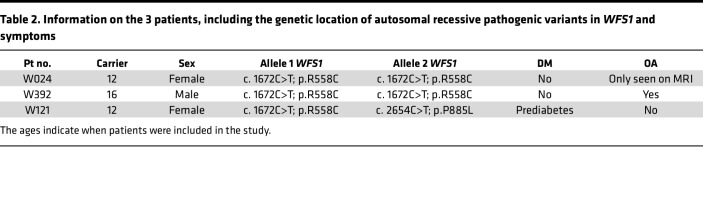
Information on the 3 patients, including the genetic location of autosomal recessive pathogenic variants in *WFS1* and symptoms

**Table 1:**
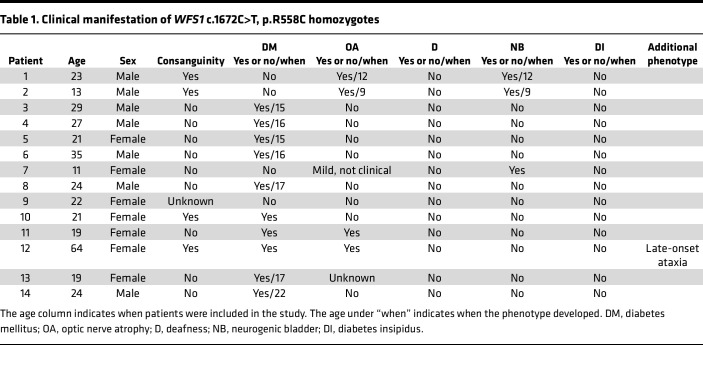
Clinical manifestation of *WFS1* c.1672C>T, p.R558C homozygotes

## Version 1 09/22/2022

Electronic publication
