# Supplementary material for: Multidimensional analysis and therapeutic development using patient iPSC–derived disease models of Wolfram syndrome
Source: JCI Insight. 2022 Sep 22;7(18):e156549. doi: 10.1172/jci.insight.156549 (PMC9675478; doi:10.1172/jci.insight.156549)
Supplement: Supplemental data [file jciinsight-7-156549-s011.pdf]

Multidimensional analysis and therapeutic development using patient iPSC-derived disease models of Wolfram syndrome

Supplementary Materials

Supplemental Methods

Supplemental References 85-91

Supplemental Figure 1. Classification of pathogenic *WFS1* variants.

Supplemental Figure 2. iPSC lines used in this study.

Supplemental Figure 3. Additional analysis of P+T effect on WFS1 expression and High-throughput screening of the NCATS Pharmaceutical Collection (NPC) to identify small molecules that increase R558C WFS1 expression.

Supplemental Figure 4. Comparison of ER stress levels in NPCs among cell lines.

Supplemental Figure 5. Comparison of each treatment effect on mitochondrial contents, mitochondrial membrane potentials and apoptosis.

Supplemental Figure 6. Comparison of each treatment effect on WFS1 protein and ER stress levels.

Supplemental Figure 7. P+T treatment reduced caspase 3/7 activity in NPCs derived from patients with typical Wolfram syndrome.

Supplemental Figure 8. Additional analyses of the patient-derived SC-islets treated with P+T.

Supplemental Figure 9. Electron microscopic (EM) analysis of SC-islets.

Supplemental Figure 10. Gene set enrichment analysis (GSEA) on the SC-β cells.

Supplemental Figure 11. Additional *in vivo* verification of a combination treatment with chemical chaperones.

Supplemental Table S1. Carrier frequencies for *WFS1* c.1672C>T, p.R558C related to Figure 1.

Supplemental Table S2. Log fold change values for key β cell genes in Fig 5B.

Supplemental Table S3. Log fold change values for *WFS1* and ER stress markers in Fig. 5C.

Supplemental Table S4. Gene Set Enrichment Analysis (GSEA) details.

Supplemental Table S5: Antibody list used in this study.

Supplemental Table S6: Primer list used in this study.

Supplemental Table S7: Media formulation, Differentiation factors and protocol for SC-islets differentiation.

Supplemental Table S8: Hashtag antibody list used in this study.

Supplemental Methods

Ashkenazi Jewish samples and Genotyping

All participants provided written consent to be used for research purposes. The consent form included that patient material would be used for clinical testing and that excess material would be de-identified for use of research purposes to characterize single gene disorders in the Ashkenazi Jewish population. We genotyped 87,093 subjects of Jewish descent for the presence/absence of the c.1672C>T p.R558C variant in the *WFS1* gene. These samples were anonymously obtained through the Dor Yeshorim screening program from 2017 to 2021. This includes samples obtained from Dor Yeshorim screening locations around the world. Subjects who use the Dor Yeshorim screening program typically represent all levels of Jewish Orthodoxy (85). When subjects submit samples for carrier screening, written consent is obtained for excess sample to be anonymously used for research to better characterize single gene disorders in the Jewish populations. Detailed methods of carrier screening at the time of testing have been previously described (86).

WFS1-HiBiT cloning, thermal stability, expression assay and high-throughput screen

The coding sequences for WT, p.R558C and p.P885L WFS1 in pDONR221 were a kind gift from Richard Suderman and David Chao in Stowers Institute for Medical Research (Kansas City, MO, USA). The coding sequences were subcloned into a pcDNA3.1(+) backbone downstream of the Gly-Ser-HiBiT-Gly-Ser tag, using InFusion PCR (Takara) as previously described (87). For the NanoLuc control vector, the coding sequence was PCR amplified from pFN31K (Promega) using forward: 5’-ACCCAAGCTGGCTAGCcaccATGGTCTTCACACTCGAAGATTTCG-3’ and reverse: 5’-GATATCTGCAGAATTCttattccatggcgatcgcgc-3’. The amplicon was gel purified and subcloned into the NheI and EcoRI sites of pcDNA3.1(+) using InFusion PCR. Thermal stability experiments were performed as previously described (33). In brief, $1x10^6$ HEK293T cells were transfected in a 6-well dish using 3 μg of plasmid and 6.25 mL of Lipofectamine 2000 per well. 48 h after transfection, cells were lifted and resuspended in CETSA

buffer containing DPBS (with $CaCl_2$ and $MgCl_2$) plus 1 g/L glucose, 1X Halt protease inhibitor cocktail. Samples were aliquoted to PCR strips at 30 μL per tube then heated for 3.5 min using a pre-heated thermal cycler, allowed to equilibrate to room temperature, and 6 μL of 6% NP40 was added to each tube. Samples were incubated at room temperature for 30 min. Substrate was added to a final concentration of 100 nM 11S fragment and 0.5X furimazine (Promega, NanoGlo Substrate). Luminescence was measured on a ViewLux HTS reader (PerkinElmer) equipped with clear filters. For temperature rescue experiments, 2.5×10^5 HEK293T cells were transfected with plasmids a 24-well plate, using Lipofectamine 2000 and OptiMEM according to the manufacturer's instructions. Cells were returned to a 37 °C incubator (5% CO2, 95% RH) for 48 h. Then, plates were left at 37 °C or shifted to 30 °C for 24 h. All medium was removed from the wells and lysis buffer (PBS + 1% NP40 + 1x protease inhibitor cocktail) was added to each well and plate was rotated at room temperature for 30 min. The lysate was pipetted to mix and 10 μL was transferred to a low volume white walled 384-well plate. 5 μL of substrate was added to a final concentration of 100 nM 11S fragment and 0.5X furimazine (Promega, NanoGlo Substrate). Luminescence was measured on a ViewLux HTS reader (PerkinElmer) equipped with clear filters. For proteasome inhibition experiments, cells were treated with 1600 nM bortezomib for 24h before measuring WFS1-HiBiT levels as described above. For P+T experiments, HEK293T cells were transfected in a T25 flask using 7.5 μg of DNA and 15 μL Lipofectamine 2000. 48 h after transfection, cells were re-seeded to white-walled 384-well plates (Corning) at 1×10^4 cells per well in 40 μL volume. After overnight incubation, cells were treated with 4-PBA and TUDCA (prepared in PBS) or vehicle control (PBS). Samples were lysed by removing all medium from wells, adding lysis buffer (PBS + 1% NP40 + 1x protease inhibitor cocktail) and rotating the plate for 30 min at room temperature. Reporter protein levels were assessed by adding 11S and furimazine to 100 nM and 0.5x, respectively. Luminescence was measured using a ViewLux HTS reader. For the high-throughput screening of the NCATS Pharmaceutical Collection, HEK293T cells were transfected with HiBiT tagged WT or R558C WFS1 (T225 flask, 62.5 μg DNA, 135 μL Lipofectamine 2000). 48 hours after transfection, cells were re-seeded to 1536-well white walled plates (Aurora, cyclic olefin polymer) at 4000 cells per well in a 5 μL volume. Cells were incubated overnight at 37 °C. Small molecules or DMSO vehicle control

were transferred (30 nL, 10 mM stock, 60 μM) using an arrayed pin tool (Wako Automation) and cells were incubated for 24 h. 1 μL of 6% NP40 solution (final concentration 1%) was added to each well and plates were incubated at room temperature for 20 min. 1 μL of substrate (final concentrations: 100 nM 11S, 0.5x furimazine) were added and luminescence was measured using a ViewLux HTS reader equipped with clear filters.

Cycloheximide chase assay and western blotting

Plasmid expressing HA-tagged human WFS1 was transfected into HeLa cells using TransIT-X2® Dynamic Delivery System (Mirus Bio; MIR 6000). After 24 hours, the cells were treated with 250 μg/ml Cycloheximide (SIGMA; C4859) then harvested at the time indicated in Figure 2E. Protein was extracted using M-PER™ Mammalian Protein Extraction Reagent (Thermo; 78501) in all experiments. An equivalent amount of total protein was loaded onto the SDS-polyacrylamide gel. Proteins were probed with primary and corresponding secondary antibodies. Antibody details can be found in Supplemental Table S5.

Actinomycin D chase assay and Real-time qPCR

The cells were treated with 5 μg/ml Actinomycin D (SIGMA, A9415) and harvested at the time indicated in Figure 2I. Total RNA was isolated using RNeasy Kits (Qiagen, 74106) and cDNA libraries were generated using high-capacity cDNA reverse transcription kits (Applied Biosystems, 4368814). Relative amounts of each transcript were calculated by the ΔΔCt method and normalized to human 18S rRNA. Quantitative PCR was performed with the Applied Biosystems ViiA7 using PowerUp™ SYBR™ Green Master Mix (Applied Biosystems, A25741). Primers used for qPCR are listed in Supplemental Table S6.

Mitochondrial respiration studies

NPCs were seeded on Seahorse cell culture plate (Agilent, 101085-004) overnight, then treated with 4-PBA and TUDCA for 48 hours. Medium was replaced with DMEM (Agilent, 103575-100) supplemented with 2.5 mM glutamine, 17.5 mM glucose and 1 mM sodium pyruvate and the plate was placed in a non-CO_2 incubator at 37℃ for 1 hour. The cell culture plate was placed in a Seahorse XFe96 Analyzer. Sequential injections of 3 µM oligomycin, 0.25 µM carbonyl cyande-4-(trifluoromethoxy) phenylhydrazone (FCCP), and 1 µM rotenone and 2 µM antimycin A were placed in the analyzer injection ports. All compounds were from a Seahorse XF Cell Mito Stress Test Kit (Agilent, 103015-100). Four OCR measurements were recorded for baseline and following each injection. Cells were lysed with 1% Triton in TE buffer and the Quant-iT Picogreen dsDNA assay kit (Invitrogen; P7589) was used to normalize OCR measurements to DNA (ng) for each well.

Quantification of mitochondrial DNA contents

Genome was extracted from NPCs using NucleoSpin Tissue (TaKaRa; 740952.5) and 10 ng DNA was used for qPCR. Mitochondrial DNA copy numbers were calculated by averaging ND1/SCLO2B1 and ND5/SERPINA1 ratio (88, 89). Primers used for qPCR are listed in Supplemental Table S6.

Measurement of mitochondrial membrane potential

NPCs were plated at 2 x 10^4 cells/well of 96-well plate. The cells were treated with each compound for 48 hours then mitochondrial membrane potential was measured using TMRM Assay Kit (Abcam; ab228569) according to the manufacturer's protocol. After the measurement, the cells were fixed with 4% PFA and stained with DAPI to determine the total cell numbers for normalization. DAPI intensity was measured at the wavelength of excitation; 350 nm and emission; 465 nm on a plate reader.

Measurement of apoptosis

1 x 10^4 NPCs or 2 x 10^4 stage 6 cells per well were seeded onto Corning® 96-well Flat Clear Bottom White Polystyrene TC-treated Microplates (Corning, 3610). After the cells were treated with 4-PBA and TUDCA, cell viability followed by caspase 3/7 activity were measured using CellTiter-Fluor™ Cell Viability Assay kit (Promega, G6080) and Caspase-Glo® 3/7 Assay System (Promega, G8090), respectively. Caspase 3/7 activity was normalized to cell viability.

Glucose stimulated insulin secretion (GSIS)

GSIS was performed similar to as we previously reported (90). Cells were collected and washed with KRB buffer (128 mM NaCl, 5 mM KCl, 2.7 mM $CaCl_2$ 1.2 mM $MgSO_4$, 1 mM Na_2HPO_4, 1.2 mM KH_2PO_4, 5 mM $NaHCO_3$, 10 mM HEPES (Gibco; 15630-080), and 0.1% BSA in water) before transfer to transwells in 2 mM glucose KRB solution for a 1 hour equilibration period. Cells were in a humidified incubator at 37°C 5% CO_2 for all incubations. Samples were transferred to another 2 mM glucose KRB solution for 1 hour and the supernatant was collected. Lastly, samples were transferred to 20 mM glucose KRB solution for 1 hour and the supernatant was collected. After all incubations, transwells were moved to TrypLE for 30 minutes at 37°C for single cell dispersion and counted for normalization.

Immunocytochemistry (ICC) and flow cytometry

For ICC of NPCs, cells were plated on eight-well chamber slides (Thermo; 177402) coated with Matrigel and laminin (5 µg/mL) and fixed in 4% Paraformaldehyde (PFA) for 20 minutes. After blocking with blocking buffer (10% donkey serum, 1% BSA and 0.1% TritonX-100 in PBS) for 1 hour, cells were incubated with primary antibodies diluted in antibody dilution buffer (1% donkey serum, 1% BSA, and 0.1% TritonX-100 in PBS) overnight at 4°C and then stained with corresponding secondary antibodies diluted in antibody dilution buffer for 1 hour at room temperature. Cells were imaged with a fluorescent microscope.

For flow cytometry, single cells were fixed with 4% PFA for 30 minutes at room temperature after single-cell dispersion with TrypLE. Cells were rinsed with PBS and underwent incubation in ICC solution (5% donkey serum and 0.1% TritonX in PBS) at room temperature for 45 minutes. After washing with ICC solution twice, cells were filtered before analysis on either the LSRII flow cytometer and BD LSR Fortessa X-20 (BD Biosciences). Dot plots and percentages for data analysis were generated with Flow Jo. Antibody details can be found in Supplemental Table S5.

Insulin content and proinsulin to insulin ratio

Clusters were placed in acid ethanol solution (1.5% HCl and 70% ethanol in milliQ water) for 72 hours at -20°C with vortexing every 24 hours. After 72 hours, samples were centrifuged for 15 minutes at 2100 RCF. Supernatant was collected and pH was neutralized with an equal volume of 1M TRIS (pH 7.5). Insulin and proinsulin levels were quantified with Human insulin ELISA (ALPCO; 80-INSHU-E10.1) and Human proinsulin ELISA (Mercodia; 10-1118-01), respectively. Proinsulin to insulin ratio was quantified by dividing proinsulin content by insulin content. Cell count was used to normalize.

Mitochondrial respiration studies with SC-islets

SC-islets were seeded on Seahorse cell culture plate (Agilent; 100777-004) overnight in ESFM. Medium is replaced with RPMI 1640 (Sigma; R1383-10XL) at 7.4 pH and plate is placed in non-CO_2 incubator at 37°C for 1 hour. Seahorse XFe24 extracellular flux analyzer (Agilent; 100777-004) and cell culture plate were placed in Seahorse XFe24 (Agilent; S7801B). Sequential injections of 3 µM oligomycin (Calbiochem), 0.25 µM carbonyl cyande-4-(trifluoromethoxy) phenylhydrazone (FCCP) (Sigma) and 1 µM rotenone (Calbiochem) and 2 µM antimycin A (Sigma) were placed in the analyzer injection ports. Four OCR measurements were recorded for baseline and following each injection. Cells were lysed with 10 mM Tris (MilliporeSigma; T6066), 1 mM EDTA (Ambion; AM9621), and 0.2% Triton X (Acros Organics; 327371000) and Quant-iT Picogreen dsDNA assay kit

(Invitrogen; P7589) was used to normalize OCR measurements to DNA (ng) for each well.

Electron microscopy

SC-islets were fixed in a modified Karnovsky's fixative of 3% glutaraldehyde and 1% paraformaldehyde in 0.1M sodium cacodylate buffer and centrifuged at 1000 rpm to a pellet. Then, the SC-islets were post-fixed in 2% osmium tetroxide in 0.1M sodium cacodylate buffer for 1 hour, en bloc stained with 3% aqueous uranyl acetate for 30 min, dehydrated in graded ethanols and embeded in PolyBed 812 (Polysciences, # 08792-1). Embedded SC-islet pellets were sectioned at 90 nm thick, post stained with Venable's lead citrate and viewed with a JEOL model 1400EX electron microscope (JEOL). Digital images were acquired using the AMT NanoSprint 12A-B (Advanced Microscopy Technology) CMOS, 12 megapixel TEM camera.

Single-cell RNA sequencing preparation

Single-cell RNA sequencing was performed similar to as we previously reported (91). Cells were single cell dispersed in TrypLE and stained with hashtags. There were four biological replicates per condition. The samples were pooled while keeping individual cell lines (W024, W121) in different samples and submitted to Washington University in St. Louis Genome Technology Access Center for library preparation and sequencing using the Chromium Single Cell 3' Library and Gel Bead Kit v3 (10x Genomics). Cells were sequenced on the NovaSeq 5000 (Illumina) at 26x98bp. For the feature library, we used custom 8bp dual same TotalSeqA-HTO indexes (TruSeqIDTdual-D807, CAGATCAT-CAGATCAT) and IDT HTO cDNA PCR additives. Hashtag antibody list is in Supplemental Table S8.

Single-cell RNA sequencing analysis

Cells with hashtags were analyzed and demultiplexed using hashtag oligos with Seurat v3.2.1. Cells with high mitochondria genes and a low number of genes mapped to the human genome were filtered out. Each sample was normalized with *NormalizeData* and *FindVariableFeatures* functions to remove outlier genes with a scaled z-score dispersion. The cells were separated into 4 groups for further analysis: W121, Ctrl; W024, Ctrl; W121, P+T; and W024, P+T. The cells were clustered on UMAP plots, where cells with similar gene expression are proximally located based on PCA with *FindNeighbors* and *FindClusters* functions. UMAP plots for each group were generated using a resolution of 0.3 and 30 dimensions to identify the separated clusters. The clusters represent different cell types which we defined based on differential gene expression (*FindAllMarkers* function) and aligning the top differentially expressed genes to the pancreatic, endocrine, exocrine, and off target cell types. To visualize the UMAP plots, we used the functions *RunUMAP* and *DimPlot*. After identifying the β cell population for each group, we found the differential expression between the β cell populations using *FindAllMarkers*. Data is represented in violin plots using *VlnPlot*.

Gene set enrichment analysis

Genes between control and treated sample groups were analyzed for Reactome and GO pathway enrichment by GSEA (4.0.2). Gene sets NES > 1.0 or < -1.0 and NOM p-val < 0.05 were considered as significantly enriched gene sets.

Animal study

129S6 whole body *Wfs1*-knockout mice were a kind gift from Dr. Sulev Kõks. 5-6 weeks-old female mice were used for this study. Food consumption rate was determined by monitoring Δ diet mass (g) per week. Animal number for each group is indicated in figure legends. Intraperitoneal glucose tolerance test (IP-GTT), intraperitoneal insulin tolerance test (IP-ITT) and *in vivo* glucose-stimulated insulin secretion test were performed according to standard procedures of the NIH-sponsored National Mouse Metabolic Phenotyping Centers

(http://www.mmpc.org). Serum insulin content was measured by rat/mouse insulin ELISA kit (EMD Millipore; EZRMI-13K).

Supplemental References

85. Ekstein J, Katzenstein H. The Dor Yeshorim story: community-based carrier screening for Tay-Sachs disease. *Adv Genet.* 2001;44:297-310.
86. Rabin R, et al. Severe epileptic encephalopathy associated with compound heterozygosity of THG1L variants in the Ashkenazi Jewish population. *American journal of medical genetics Part A.* 2021;185(5):1589-97.
87. Sanchez TW, et al. High-Throughput Detection of Ligand-Protein Binding Using a SplitLuc Cellular Thermal Shift Assay. *Methods in molecular biology.* 2021;2365:21-41.
88. Yu Y, et al. Hepatocyte-like cells differentiated from human induced pluripotent stem cells: relevance to cellular therapies. *Stem Cell Res.* 2012;9(3):196-207.
89. Augustyniak J, et al. Mitochondrial biogenesis and neural differentiation of human iPSC is modulated by idebenone in a developmental stage-dependent manner. *Biogerontology.* 2017;18(4):665-77.
90. Velazco-Cruz L, et al. Acquisition of Dynamic Function in Human Stem Cell-Derived beta Cells. *Stem Cell Reports.* 2019;12(2):351-65.
91. Augsornworawat P, et al. Single-Cell Transcriptome Profiling Reveals beta Cell Maturation in Stem Cell-Derived Islets after Transplantation. *Cell reports.* 2020;32(8):108067.

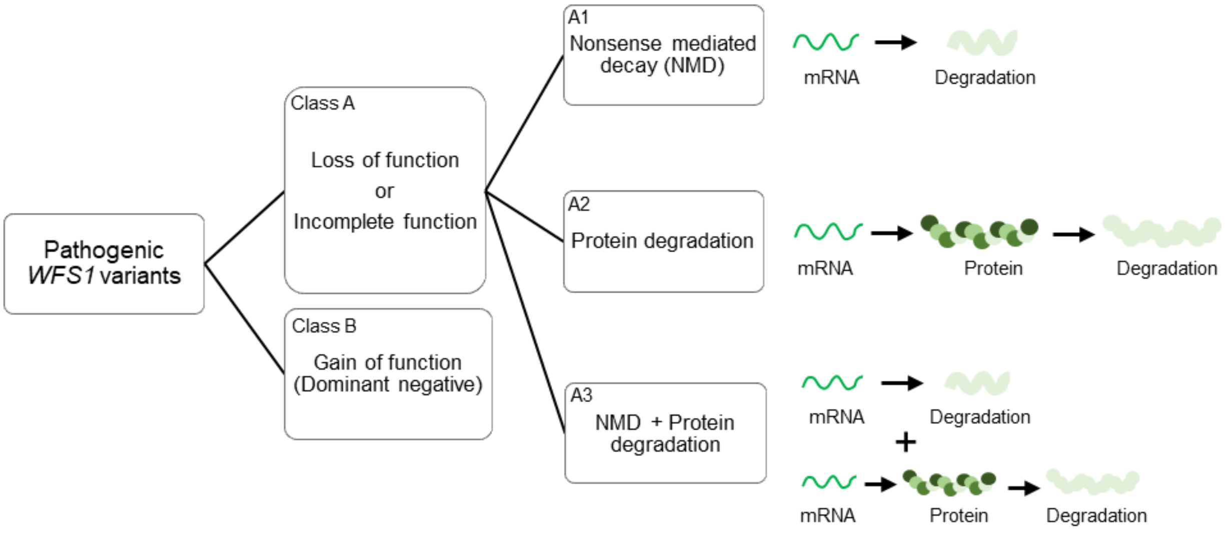

Supplemental Figure 1. Classification of pathogenic *WFS1* variants.

A schematic of classification of pathogenic *WFS1* variants in terms of protein expression.

A

iPSC line	At a.a. position R558			At a.a. position P885		
	Total Reads	c.1672C>T	WT	Total Reads	c.2654C>T	WT
W024	3994	100%	0	5999	0	100%
W392	5426	100%	0	4687	0	100%
W121	4174	50%	50%	5973	50%	50%

B

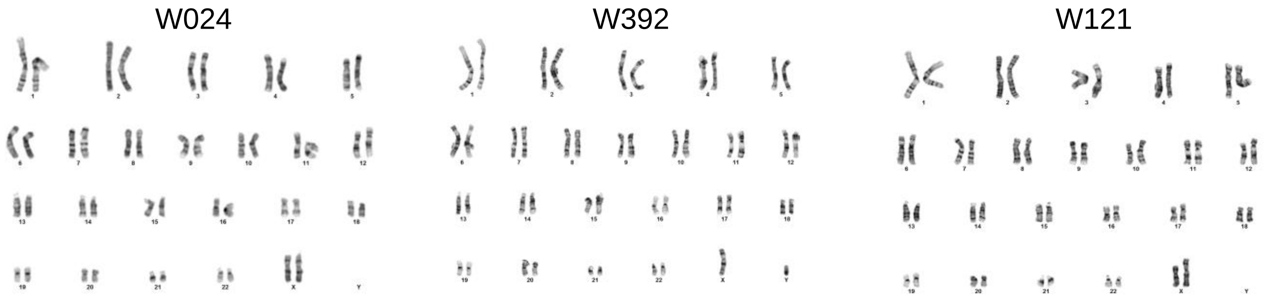

C

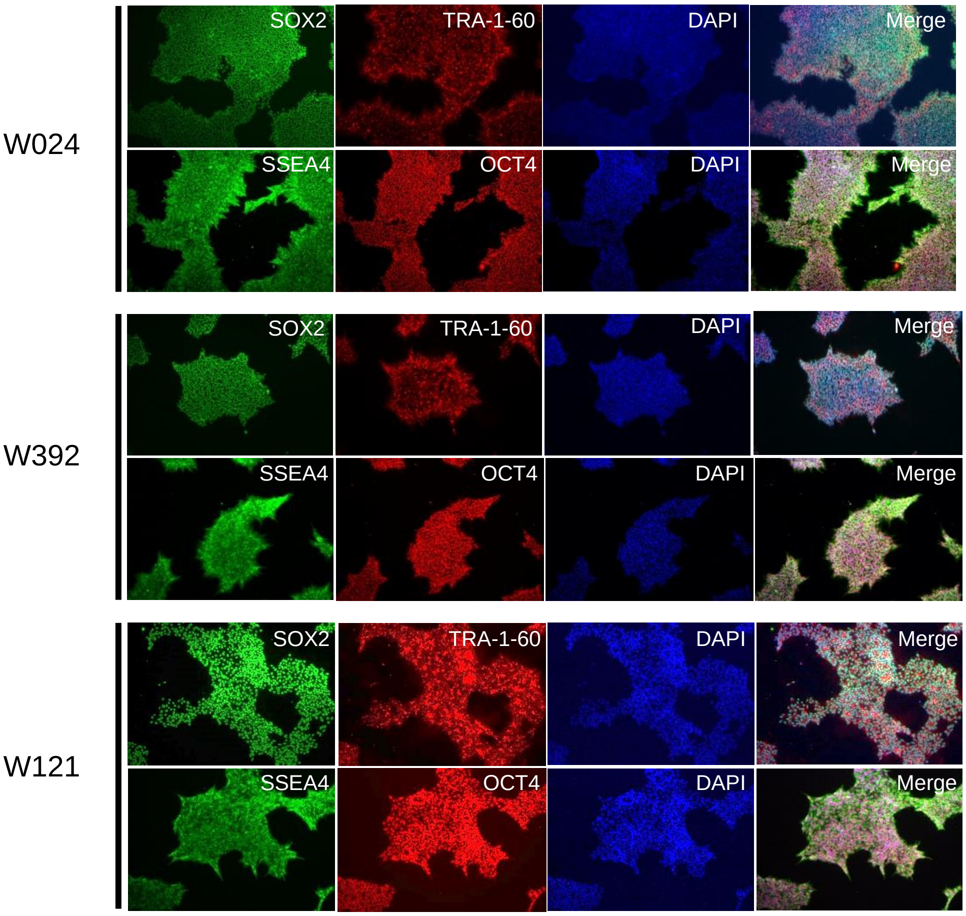

Supplemental Figure 2. iPSC lines used in this study.

(A) Confirmation of the mutations in *WFS1* gene in iPSC lines derived from patients with Wolfram syndrome. (B) Normal 46XX (W024 and W121) and 46XY (W392) karyotypes of iPSC lines. (C) Immunofluorescence staining of iPSC markers.

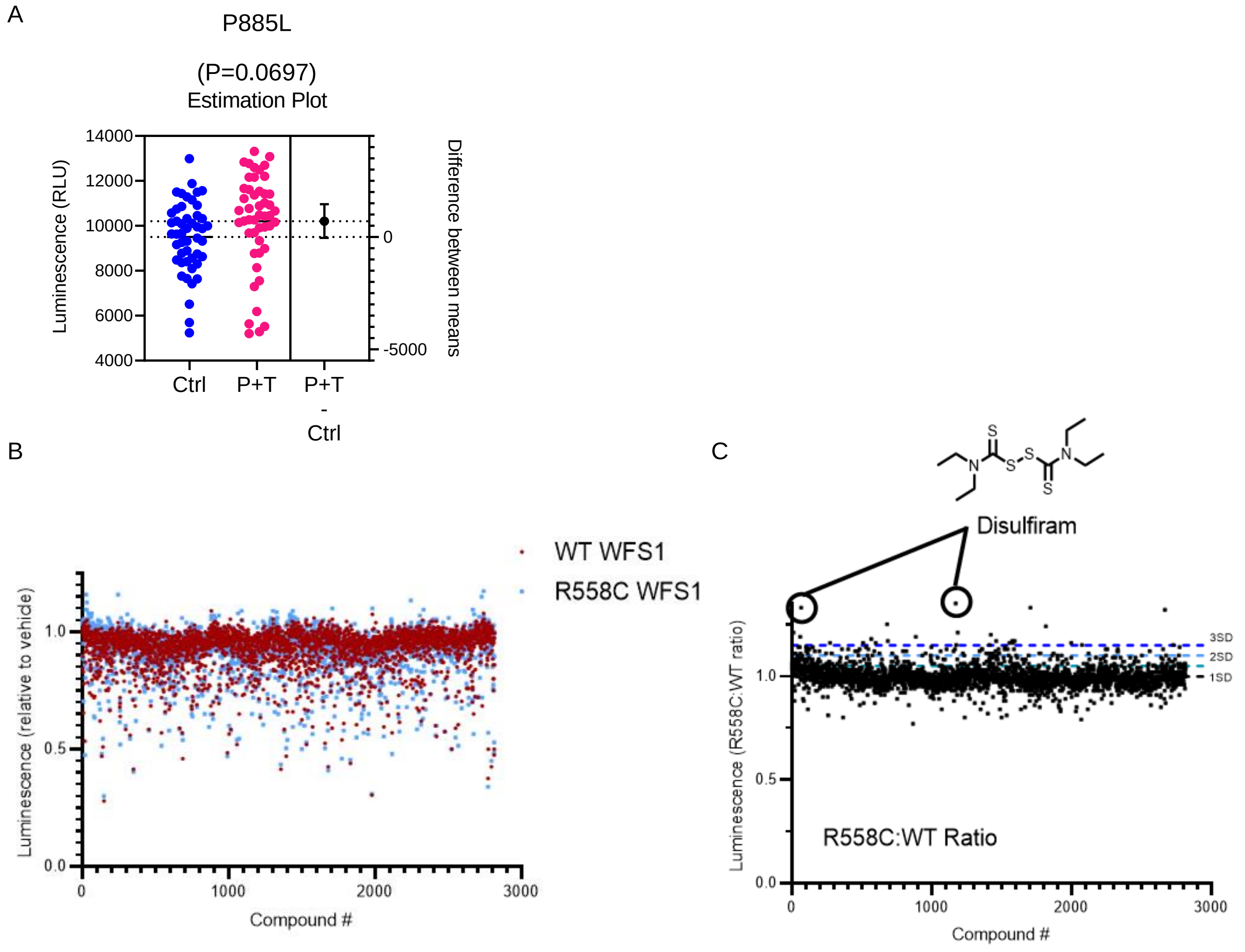

Supplemental Figure 3. Additional analysis of P+T effect on WFS1 expression and High-throughput screening of the NCATS Pharmaceutical Collection (NPC) to identify small molecules that increase R558C WFS1 expression.

(A) Expression of HiBiT tagged WFS1 p.P885L after treatment with 500 μM 4-PBA and 50 μM TUDCA (P+T) for 24 hours (n=48, the p-value by unpaired t-test). (B) Expression of HiBiT tagged WFS1 variants after treatment with compounds (60 μM) for 24 hours. The effect of each compound for WT (red dot) and R558C (blue dot) WFS1 proteins is shown. The library does not include TUDCA. (C) R558C selectivity was assessed by calculating the ratiometric effect for R558C relative to WT WFS1. Disulfiram, was the top hit (two samples contained in the library).

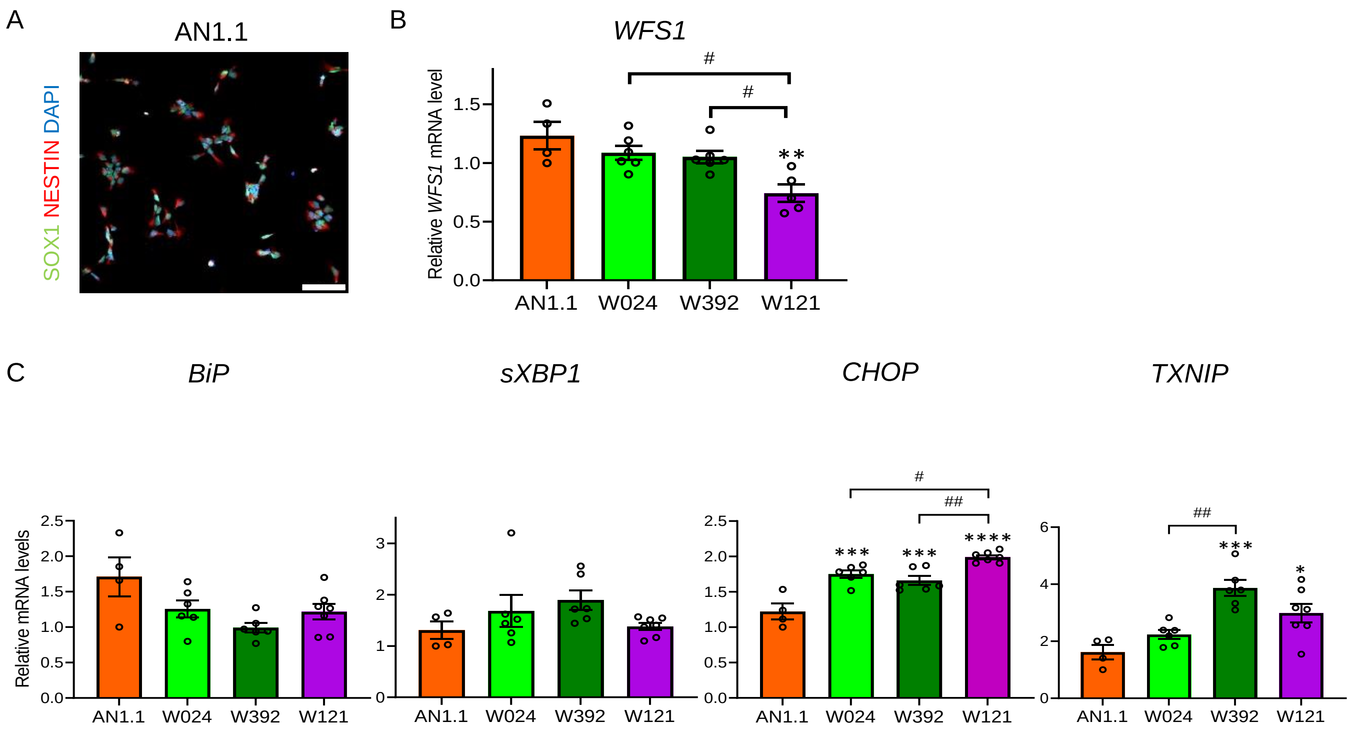

Supplemental Figure 4. Comparison of ER stress levels in NPCs among cell lines.

(A) Representative immunofluorescence image of neural progenitor cell (NPC) markers in NPCs differentiated from control iPSC line AN1.1. Scale bar: 100 μm. (B) qPCR analysis of *WFS1* expression in NPCs differentiated from each iPSC line (AN1.1: n=4, W024 and W392: n=6, W121: n=7. *P<0.05, ***P<0.001 and ****P<0.0001 by one-way ANOVA compared to AN1.1. #P<0.05 and ##P<0.01 by one-way ANOVA). (C) qPCR analysis of ER stress related genes in NPCs differentiated from each iPSC line. (AN1.1: n=4, W024 and W392: n=6, W121: n=7. *P<0.05, ***P<0.001 and ****P<0.0001 by one-way ANOVA compared to AN1.1. #P<0.05 and ##P<0.01 by one-way ANOVA).

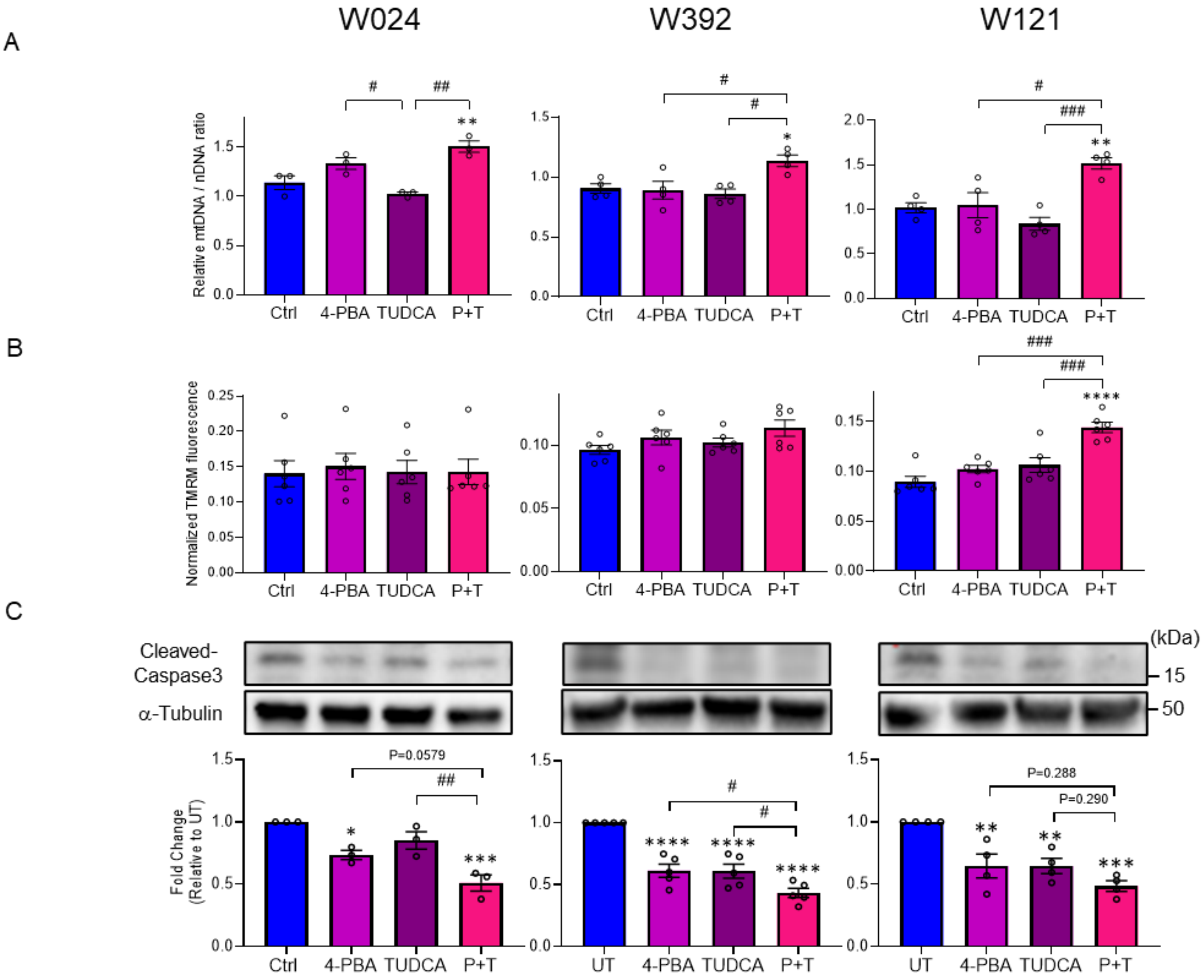
W024
W392
W121
A
B
C
Relative mtDNA / nDNA ratio
Normalized TMRM fluorescence
Ctrl
4-PBA
TUDCA
P+T
UT
Cleaved-
Caspase3
α-Tubulin
(kDa)
15
50
Fold Change
(Relative to UT)
P=0.0579
P=0.288
P=0.290

Supplemental Figure 5. Comparison of each treatment effect on mitochondrial DNA contents, mitochondrial membrane potentials and apoptosis.

(A) Relative mitochondrial DNA (mtDNA) copy numbers normalized to nuclear DNA (nDNA) measured by qPCR analysis in NPCs treated with or without 4-PBA, TUDCA or P+T for 48 hours. (W024: n=3, W392: n=4, W121: n=4. *P<0.05 and **P<0.01 by one-way ANOVA compared to Ctrl. #P<0.05, ##P<0.01 and ###P<0.001 by one-way ANOVA). (B) Mitochondrial membrane potentials measured by fluorescent probe TMRM in NPCs treated with or without 4-PBA, TUDCA or P+T for 48 hours (n=6. ****P<0.0001 by one-way ANOVA compared to Ctrl. ###P<0.001 by one-way ANOVA). (C) (Upper) Representative blotting image of cleaved-Caspase3 and α-Tubulin in NPCs treated with or without 4-PBA, TUDCA or P+T for 48 hours. (Lower) A quantification of cleaved-Caspase3 protein levels normalized with α-Tubulin. (n=3, *P<0.05, **P<0.01, ***P<0.001 and ****P<0.0001 by one-way ANOVA compared to Ctrl. #P<0.05 and ##P<0.01 by one-way ANOVA).

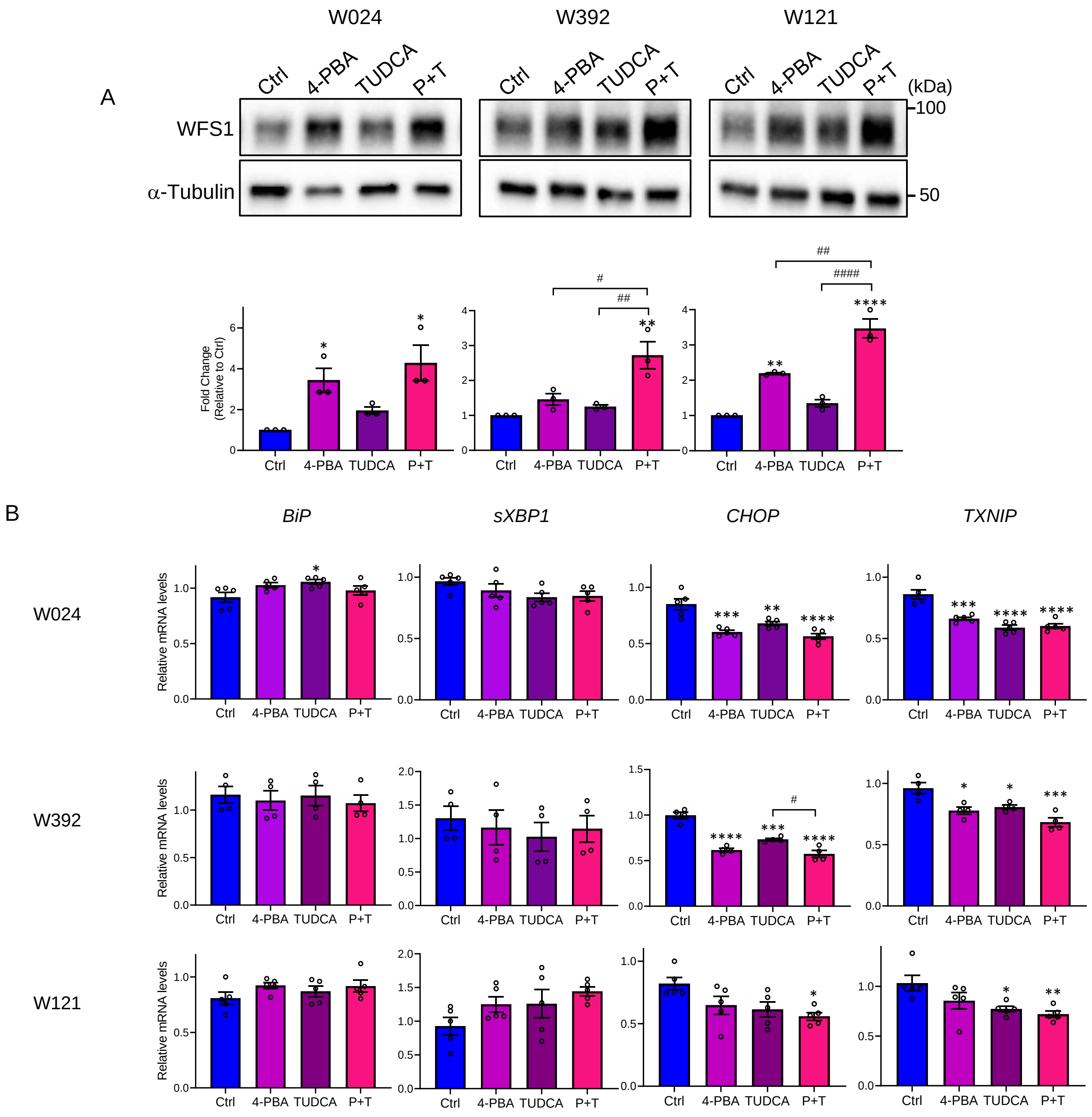

A
W024
W392
W121
Ctrl
4-PBA
TUDCA
P+T
(kDa)
WFS1
α-Tubulin
100
50
Fold Change (Relative to Ctrl)
B
BiP
sXBP1
CHOP
TXNIP
W024
W392
W121
Relative mRNA levels

Supplemental Figure 6. Comparison of each treatment effect on WFS1 protein and ER stress levels.

(A) (Upper) Representative blotting image of WFS1 and α-Tubulin in iPSCs treated with or without 4-PBA, TUDCA or P+T for 48 hours. (Lower) A quantification of WFS1 protein levels normalized with α-Tubulin. (n=3, *P<0.05, **P<0.01 and ****P<0.0001 by one-way ANOVA compared to Ctrl. #P<0.05, ##P<0.01 and ####P<0.0001 by one-way ANOVA).

(B) qPCR analysis of ER stress related genes in NPCs treated with or without 4-PBA, TUDCA or P+T. (W024: n=5, W392: n=4, W121: n=5. *P<0.05, **P<0.01, ***P<0.001 and ****P<0.0001 by one-way ANOVA compared to Ctrl. #P<0.05 by one-way ANOVA).

A

Pt #	Age	Sex	Allele 1 *WFS1*	Allele 2 *WFS1*	DM	OA
W4	23.8	Female	c.1112G>A; p.W371X	c.1885C>T; p.R629W	2.3	5
W9	14.3	Male	c.376G>A; p.A126T	c.1838G>A; p.W613X	10.8	11
W13	5.9	Female	c.599delT; p.L200fs286Stop	c.2254G>T; p.E752Stop	4.8	5.2
W15	10.8	Female	c.439delC; p.R147fsX163	c.1620G>A; p.W540X	2.8	7

B

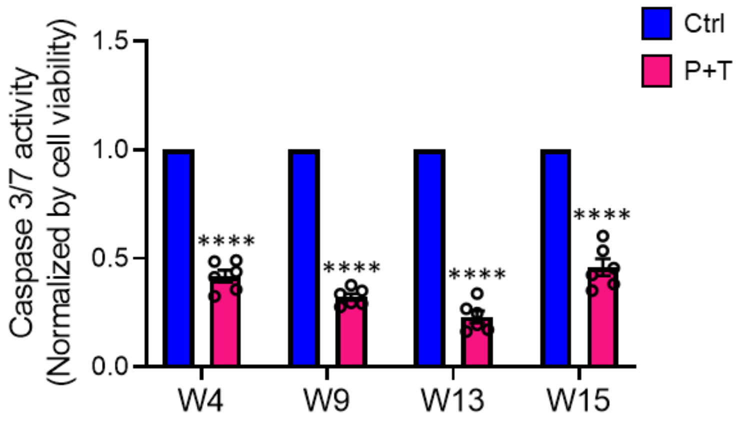

Supplemental Figure 7. P+T treatment reduced caspase 3/7 activity in NPCs derived from patients with typical Wolfram syndrome.

(A) Information on the four patients with typical Wolfram syndrome, including the genetic location of autosomal recessive pathogenic variants in *WFS1* and the onset age of symptoms. The ages indicate when subjects were included in the study. DM: Diabetes Mellitus, OA: Optic nerve atrophy. (B) Caspase 3/7 activity normalized by cell viability in NPCs treated with or without P+T for 24 hours. (n=6, ****P < 0.0001 by unpaired t-test compared to Ctrl).

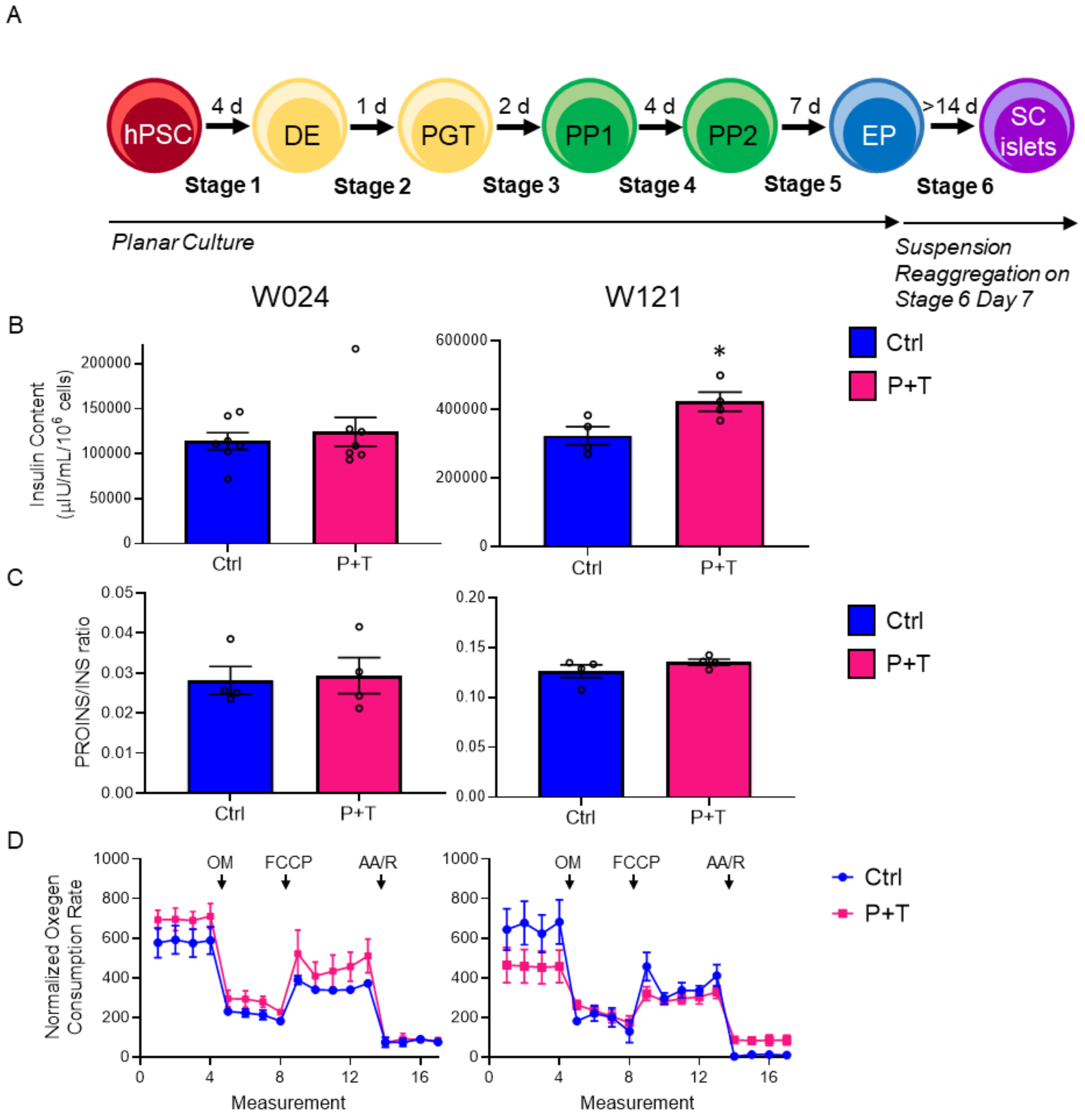
A
hPSC
4 d
DE
1 d
PGT
2 d
PP1
4 d
PP2
7 d
EP
>14 d
SC islets
Stage 1
Stage 2
Stage 3
Stage 4
Stage 5
Stage 6
Planar Culture
Suspension Reaggregation on Stage 6 Day 7
W024
W121
B
Insulin Content (µIU/mL/10^6 cells)
Ctrl
P+T
*
C
PROINS/INS ratio
D
Normalized Oxegen Consumption Rate
OM
FCCP
AA/R
Measurement

Supplemental Figure 8. Additional analyses of the patient-derived SC-islets treated with P+T.

(A) A schematic of 6 stage SC-islets differentiation protocol mimicking embryonic development of pancreatic endocrine cells. hPSC: Human pluripotent stem cell, DE: Definitive endoderm, PGT: Pancreatic gut tube, PP1: Pancreatic progenitor 1, PP2: Pancreatic progenitor 2, EP: Endocrine progenitor, SC-islets: Stem cell-derived islets. (B) Insulin content of the patient-derived SC-islets treated with or without P+T for 7 days (W024: n=7, W121: n=4. *$P<0.05$ by unpaired t-test compared to Ctrl). (C) The ratio of proinsulin and insulin content of the patient-derived SC-islets treated with or without P+T for 7 days (W024: n=4, W121: n=4). (D) Mitochondrial respiration of the patient-derived SC-islets treated with or without P+T for 7 days. Respiration was interrogated by measuring changes in relative oxygen consumption rate (OCR) after injection with oligomycin (OM), FCCP, and antimycin A (AA)/rotenone (R) (W024: n=3, W121: n=3).

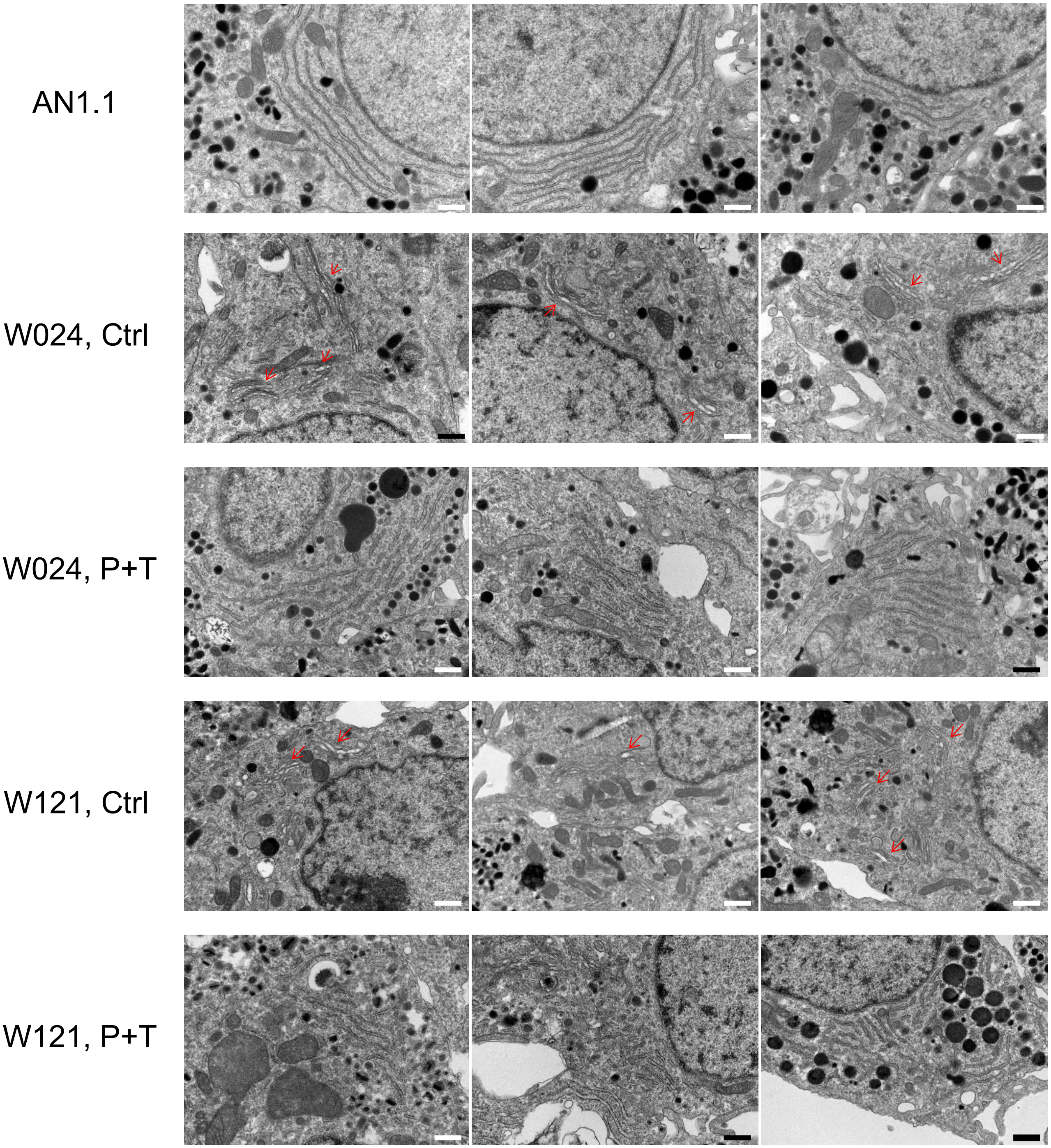

Supplemental Figure 9. Electron microscopic (EM) analysis of SC-islets.

Representative EM images for AN1.1, W024 and W121 stage 6 SC-islets treated with or without P+T for 7 days. Scale bar, 600 nm. Red arrows indicate dilated endoplasmic reticulum (ER).

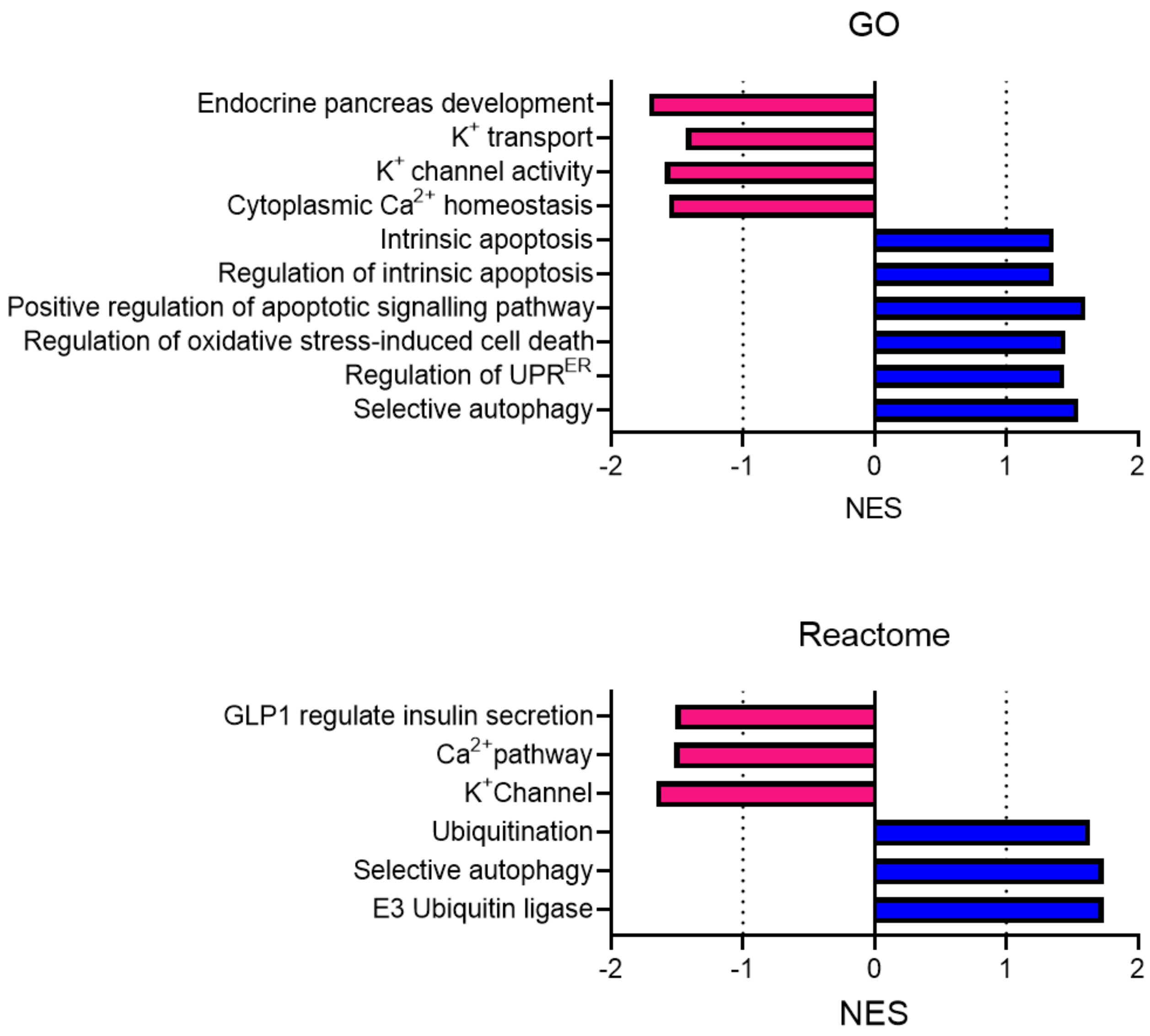

Supplemental Figure 10. Gene set enrichment analysis (GSEA) on the SC-β cells.

GO and Reactome GSEA, quantified by the normalized enrichment score (NES), for pathways upregulated in the combined population of W024 and W121 SC-β cells treated with (red) or without (blue) P+T. NES values, P values, FDR q-values, and gene set lists are available in Supplemental Table S4.

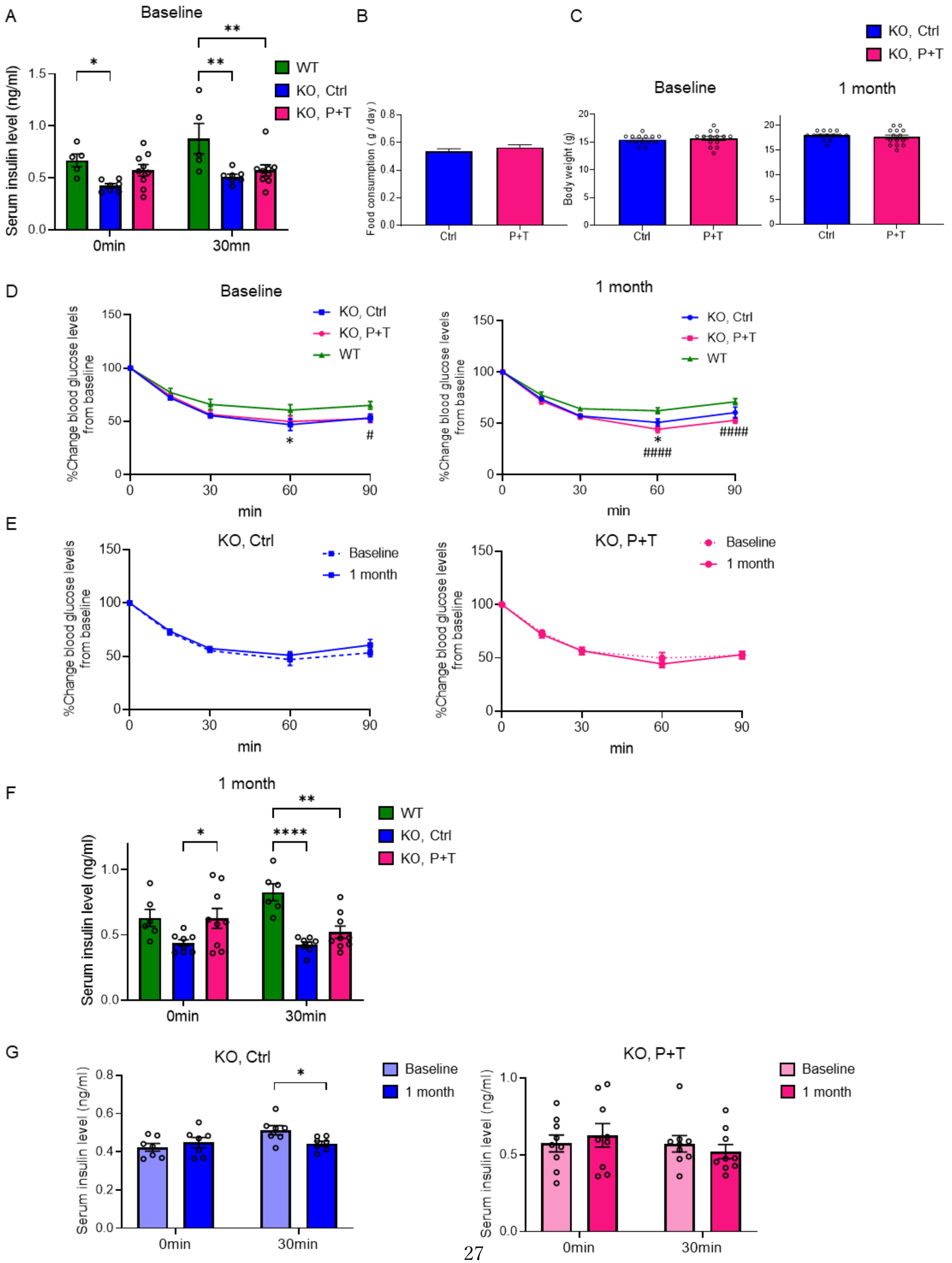
A
Baseline
Serum insulin level (ng/ml)
WT
KO, Ctrl
KO, P+T
0min
30mn
B
Food consumption (g / day)
Ctrl
P+T
C
KO, Ctrl
KO, P+T
Baseline
1 month
Body weight (g)
D
Baseline
1 month
%Change blood glucose levels from baseline
min
E
KO, Ctrl
KO, P+T
Baseline
1 month
F
1 month
0min
30min
G
KO, Ctrl
KO, P+T
Serum insulin level (ng/ml)
Baseline
1 month
0min
30min

Supplemental Figure 11. Additional *in vivo* verification of a combination treatment with chemical chaperones.

(A) Serum insulin levels in WT and *Wfs1* KO mice at 5-6 weeks old before feeding with either Ctrl or P+T chow (WT: n=5, KO, Ctrl: n=7, KO, P+T: n=9. *$P < 0.05$ and **$P < 0.01$ by two-way ANOVA). (B) Food consumption rate in *Wfs1* KO mice fed with either Ctrl or P+T chow. (C) Body weight of *Wfs1* KO mice before and after feeding with either Ctrl or P+T chow (KO, Ctrl: n=14, KO, P+T: n=14). (D) IP-ITT with WT or *Wfs1* KO mice before (Baseline) and after (1 month) feeding with either Ctrl or P+T chow (WT: n=5, KO, Ctrl: n=5, KO, P+T: n=8. *$P<0.05$ by two-way ANOVA compared between WT and KO, Ctrl. #$P<0.05$ and ####$P<0.0001$ by two-way ANOVA compared between WT and KO, P+T). (E) IP-ITT with *Wfs1* KO mice fed with either Ctrl or P+T chow comparing between before (Baseline) and after (1 month) the feeding for each group. (F) Serum insulin levels in WT and *Wfs1* KO mice fed with either Ctrl or P+T chow for 1 month (WT: n=6, KO, Ctrl: n=7, KO, P+T: n=9. *$P<0.05$, **$P<0.01$ and ****$P<0.0001$ by two-way ANOVA). (G) Serum insulin levels in *Wfs1* KO mice fed with either Ctrl or P+T comparing between before (Baseline) and after (1 month) the feeding for each group (*$P<0.05$ by paired t-test).

Supplemental Table S1. Carrier frequencies for *WFS1* c.1672C>T, p.R558C related to Figure 1.

A. Genotype counts and carrier frequencies for *WFS1* c.1672C>T, p.R558C by main ancestry categories

Ancestry	POS	NEG	Total	Frequency in %	Frequency
Ashkenazi	1435	60500	61935	2.32%	1 in 43
Ashkenazi/Sephardi	142	10635	10777	1.32%	1 in 76
Convert	8	675	683	1.17%	1 in 85
Sephardi	6	13602	13608	0.04%	1 in 2268
Unknown	4	86	90	4.44%	1 in 23

B. Genotype counts and carrier frequency of *WFS1* c.1672C>T, p.R558C by country of grandparental origin

Country	Carrier	Non-carrier	Total	Frequency in %	Frequency
Romania	9	248	257	3.50%	1 in 29
Poland	62	2350	2412	2.57%	1 in 39
Russia	19	897	916	2.07%	1 in 48
Hungary	85	5131	5216	1.63%	1 in 61
Germany	3	185	188	1.60%	1 in 63
Lithuania	2	229	231	0.87%	1 in 116

Supplemental Table S2. Log fold change values for key β cell genes in Fig 5B.

A.

Gene	W024 Ctrl vs W121 Ctrl					
	p_val	avg_logFC	pct.1	pct.2	p_val_adj	Higher
INS	0.0043296	-0.224086	1	1	1	W121
ISL1	0.0107986	0.1615993	0.936	0.905	1	W024
NKX6-1	0.0002553	0.1802035	0.548	0.401	1	W024
NKX2-2	0.034348	0.1023453	0.599	0.541	1	W024
GCK	0.1084081	-0.037919	0.312	0.395	1	W121
PDX1	6.36E-05	0.1810824	0.631	0.488	1	W024
MT1X	0.8951347	-0.119034	0.153	0.146	1	W121
ERO1B	1.48E-02	0.1774299	0.879	0.847	1	W024
SLC30A8	2.761E-10	0.4252099	0.796	0.631	1.01E-05	W024

B.

Gene	W024 Ctrl vs W024 P+T					
	p_val	avg_logFC	pct.1	pct.2	p_val_adj	Higher
INS	0.0887428	-0.139229	1	1	1	P+T
CHGA	0.0113484	-0.130997	0.994	1	1	P+T
ISL1	0.5811342	-0.022683	0.936	0.893	1	P+T
NKX6-1	0.2393752	-0.117362	0.548	0.579	1	P+T
NKX2-2	0.8340094	-0.031459	0.599	0.586	1	P+T
GCK	0.815778	-0.010663	0.312	0.329	1	P+T
PDX1	0.1236773	-0.139287	0.631	0.65	1	P+T
MT1X	0.0009771	-0.315673	0.153	0.3	1	P+T
ERO1B	7.52E-06	-0.278009	0.879	0.921	0.2752081	P+T
SLC30A8	0.7489582	-0.064865	0.796	0.807	1	P+T

C.

Gene	W121 Ctrl vs W121 P+T					
	p_val	avg_logFC	pct.1	pct.2	p_val_adj	Higher
INS	0.0100539	-0.09245	1	1	1	P+T
CHGA	0.5976865	0.0189148	0.996	0.994	1	UT
ISL1	0.9888308	-0.012851	0.905	0.899	1	P+T
NKX6-1	0.1400016	-0.03988	0.401	0.437	1	P+T
NKX2-2	0.2757188	-0.058009	0.541	0.546	1	P+T
GCK	0.0472464	0.0415454	0.395	0.346	1	UT
PDX1	0.6208715	-0.021635	0.488	0.484	1	P+T
MT1X	4.67E-12	-0.229312	0.146	0.296	1.71E-07	P+T
ERO1B	1.59E-08	-0.201234	0.847	0.9	0.0005837	P+T
SLC30A8	0.0186384	-0.09825	0.631	0.687	1	P+T

Log fold change values between (A) W024 and W121 SC-β cells, (B) Ctrl and P+T treated W024 SC-β cells, and (C) Ctrl and P+T treated W121 SC-β cells, detailing which cell types has greater expression according to avg_log FC. Nonadjusted (p_val) and adjusted (p_val_adj) p-values, and percentage of cells positive for measured gene in (A) W024 (pct. 1) and W121 (pct. 2) SC-β cells, (B) Ctrl and P+T treated W024 SC-β cells, and (C) Ctrl and P+T treated W121 SC-β cells calculated for violing plots in Fig. 5B.

Supplemental Table S3. Log fold change values for *WFS1* and ER stress markers in Fig. 5C.

A.

Gene	W024 Ctrl vs W121 Ctrl					
	p_val	avg_logFC	pct.1	pct.2	p_val_adj	Higher
WFS1	0.1444364	0.0607617	0.331	0.276	1	W024
DDIT3/CHOP	0.0201864	-0.158648	0.401	0.493	1	W121
TXNIP	3.07E-08	-0.33692	0.268	0.493	0.0011245	W121
ATF4	0.0570583	-0.112571	0.631	0.688	1	W121
TRIB3	0.0653016	-0.094221	0.217	0.287	1	W121
PPP1R15A/GADD34A	0.0252822	-0.179302	0.178	0.254	1	W121
CASP3	0.1065682	-0.057606	0.261	0.341	1	W121
HERPUD1	4.74E-06	-0.404152	0.707	0.802	0.1735921	W121
HSPA5/BiP	1.67E-11	-0.513738	0.955	0.984	6.10E-07	W121
ATF6	0.7096015	-0.020428	0.408	0.436	1	W121

B.

Gene	W024 Ctrl vs W024 P+T					
	p_val	avg_logFC	pct.1	pct.2	p_val_adj	Higher
WFS1	0.0043594	-0.128791	0.331	0.471	1	P+T
DDIT3/CHOP	0.0202896	0.1821288	0.401	0.271	1	UT
TXNIP	0.7942256	0.0254944	0.268	0.271	1	UT
ATF4	0.2174107	-0.024214	0.631	0.671	1	P+T
TRIB3	0.927267	-0.01233	0.217	0.214	1	P+T
PPP1R15A/GADD34A	0.0052629	0.1027399	0.178	0.071	1	UT
CASP3	0.6787669	0.0389381	0.261	0.243	1	UT
HERPUD1	0.0189208	0.1433239	0.707	0.593	1	UT
HSPA5/BiP	2.65E-06	-0.298749	0.955	0.986	0.0970356	P+T
ATF6	0.421929	0.0187674	0.408	0.35	1	UT

C.

Gene	W121 Ctrl vs W121 P+T					
	p_val	avg_logFC	pct.1	pct.2	p_val_adj	Higher
WFS1	0.6468288	-0.027072	0.276	0.284	1	P+T
DDIT3/CHOP	9.79E-05	0.1507199	0.493	0.394	1	UT
TXNIP	0.1150373	0.0620431	0.493	0.451	1	UT
ATF4	0.190563	0.0520772	0.688	0.653	1	UT
TRIB3	4.43E-05	0.0911685	0.287	0.196	1	UT
PPP1R15A/GADD34A	0.00017	0.1662685	0.254	0.174	1	UT
CASP3	0.0988933	0.0277898	0.341	0.29	1	UT
HERPUD1	0.8180927	0.0634322	0.802	0.772	1	UT
HSPA5/BiP	0.000345	-0.029453	0.984	0.974	1	P+T
ATF6	0.8640454	-0.017208	0.436	0.426	1	P+T

Log fold change values between (A) W024 and W121 SC-β cells, (B) Ctrl and P+T treated W024 SC-β cells, and (C) Ctrl and P+T treated W121 SC-β cells, detailing which cell types has greater expression according to avg_log FC. Nonadjusted (p_val) and adjusted (p_val_adj) p-values, and percentage of cells positive for measured gene in (A) W024 (pct. 1) and W121 (pct. 2) SC-β cells, (B) Ctrl and P+T treated W024 SC-β cells, and (C) Ctrl and P+T treated W121 SC-β cells calculated for violin plots in Fig. 5C.

Supplemental Table S4. Gene Set Enrichment Analysis (GSEA) details.

A. Gene Ontology (GO) Pathways

Enriched in Ctrl compared to P+T

Category	Name in Paper	GSEA Pathway Name	NES	NOM p-val	FDR q-val
Inflammation	Response to IL-12	GOBP_RESPONSE_TO_INTERLEUKIN_12	1.94	0	0.005
	non-canonical NF-kB signal	GOBP_NIK_NF_KAPPAB_SIGNALING	1.62	0.002	0.059
NMD	Nonsense mediated decay	GOBP_NUCLEAR_TRANSCRIBED_MRNA_CATABOLIC_PROCESS_NONSENSE_MEDIATED_DECAY	1.87	0	0.008
Protein degradation	Ubiquitin-dependent protein degradation	GOBP_REGULATION_OF_UBIQUITIN_DEPENDENT_PROTEIN_CATABOLIC_PROCESS	1.67	0	0.043
Selective autophagy	Selective autophagy	GOBP_SELECTIVE_AUTOPHAGY	1.55	0.006	0.088
	Mitophagy	GOBP_AUTOPHAGY_OF_MITOCHONDRION	1.35	0.048	0.229
ER stress	ER stress response	GOBP_RESPONSE_TO_ENDOPLASMIC_RETICULUM_STRESS	1.44	0.006	0.163
	Regulation of UPR^{ER}	GOBP_REGULATION_OF_ENDOPLASMIC_RETICULUM_UNFOLDED_PROTEIN_RESPONSE	1.44	0.049	0.164
Oxidative stress	Regulation of oxidative stress-induced cell death	GOBP_REGULATION_OF_OXIDATIVE_STRESS_INDUCED_CELL_DEATH	1.45	0.033	0.154
Apoptosis	Positive regulation of apoptotic signaling pathway	GOBP_POSITIVE_REGULATION_OF_APOPTOTIC_SIGNALING_PATHWAY	1.6	0	0.066
	Apoptotic signaling pathway	GOBP_APOPTOTIC_SIGNALING_PATHWAY	1.48	0	0.131
	Regulation of intrinsic apoptosis	GOBP_REGULATION_OF_INTRINSIC_APOPTOTIC_SIGNALING_PATHWAY	1.36	0.045	0.221
	Intrinsic apoptosis	GOBP_INTRINSIC_APOPTOTIC_SIGNALING_PATHWAY	1.36	0.044	0.228

Enriched in P+T compared to Ctrl

Category	Name in Paper	GSEA Pathway Name	NES	NOM p-val	FDR q-val
Regulatoin of Ca^{2+} level	Cytosolic Ca2+ homeostasis	GOBP_REGULATION_OF_CYTOSOLIC_CALCIUM_ION_CONCENTRATION	-1.56	0	0.148
Regulation of K^+ level	K^+ channel activity	GOMF_POTASSIUM_CHANNEL_ACTIVITY	-1.59	0	0.142
	K^+ transport	GOBP_POTASSIUM_ION_TRANSPORT	-1.43	0.01	0.212
β cell development	Endocrine pancreas development	GOBP_ENDOCRINE_PANCREAS_DEVELOPMENT	-1.71	0.004	0.107
	Pancreas development	GOBP_PANCREAS_DEVELOPMENT	-1.61	0.002	0.144

B. Reactome Pathways

Enriched in Ctrl compared to P+T

Category	Name in Paper	GSEA Pathway Name	NES	NOM p-val	FDR q-val
Inflammation	JAK-STAT signaling	REACTOME_GENE_AND_PROTEIN_EXPRESSION_BY_JAK_STAT_SIGNALING_AFTER_INTERLEUKIN_12_STIMULATION	1.96	0	0
	IL-12 signaling	REACTOME_INTERLEUKIN_12_SIGNALING	1.95	0	0.001
	Non-canonical NF-kB pathway	REACTOME_TNFR2_NON_CANONICAL_NF_KB_PATHWAY	1.61	0.004	0.027
	TNF signaling	REACTOME_TNF_SIGNALING	1.59	0.012	0.031
	IL-1 signaling	REACTOME_INTERLEUKIN_1_SIGNALING	1.57	0.008	0.036
NMD	Nonsense mediated decay	REACTOME_NONSENSE_MEDIATED_DECAY_NMD	1.84	0.002	0.003
Ubiquitination-mediated protein degradation	E3 Ubiquitin ligase	REACTOME_E3_UBIQUITIN_LIGASES_UBIQUITINATE_TARGET_PROTEINS	1.74	0	0.01
	Ubiquitination	REACTOME_PROTEIN_UBIQUITINATION	1.64	0.004	0.022
Selective autophagy	Selective autophagy	REACTOME_SELECTIVE_AUTOPHAGY	1.74	0	0.01
	Autophagy	REACTOME_AUTOPHAGY	1.74	0	0.01
	Mitophagy	REACTOME_MITOPHAGY	1.64	0.002	0.021

Enriched in P+T compared to Ctrl

Category	Name in Paper	GSEA Pathway Name	NES	NOM p-val	FDR q-val
Regulation of K+ level	K^+ Channel	REACTOME_POTASSIUM_CHANNELS	-1.66	0.002	0.105
Regulatoin of Ca^{2+} level	Ca^{2+} pathway	REACTOME_CA2_PATHWAY	-1.52	0.01	0.26
Insulin secretion	GLP1 regulate insulin secretion	REACTOME_GLUCAGON_LIKE_PEPTIDE_1_GLP1_REGULATES_INSULIN_SECRETION	-1.51	0.014	0.254
	Insulin secretion	REACTOME_REGULATION_OF_INSULIN_SECRETION	-1.47	0.01	0.308
β cell development	β cell development	REACTOME_REGULATION_OF_BETA_CELL_DEVELOPMENT	-1.5	0.03	0.27

(A-B) (A) GO and (B) Reactome GSEA details. Gene sets with NES > 1.0 or < -1.0 and NOM p-val < 0.05 were considered as significantly enriched gene sets. NES: normalized enrichment score, NOM p-val: normalized p-value, and FDR q-val: False discovery rate q-value.

Supplemental Table S5: Antibody list used in this study.

**Antibody**	**Company**	**Catalog #**	**Dilution**
C-Peptide	DSHB	GN-ID4-S	1:300
CHGA	Abcam	Ab15160	1:100
WFS1	CST	8749	1:1000
NKX6-1	DSHB	F55A12-S	1:100
HA	CST	3724	1:1000
α-Tubulin	CST	2125	1:1000
Sox1	R&D	AF3369	1:200
Nestin	SIGMA	N5413	1:200
Cleaved-Caspase3	CST	9664	1:1000

Supplemental Table S6: Primer list used in this study.

Primer	Forward	Reverse
BiP	TGTTCAACCAATTATCAGCAAACTC	TTCTGCTGTATCCTCTTCACCAGT
CHOP	AGAACCAGGAAACGGAAACAG	TCTCCTTCATGCGCTGCTTT
sXBP1	CTGAGTCCGAATCAGGTGCAG	ATCCATGGGGAGATGTTCTGG
TXNIP	AGGAAGCTCAAAGCCGAACT	ACGCTTCTTCTGGAAGACCA
WFS1	GAGCCCTGAGGACCTGCC	TCTCCATGATGGCGTGCA
18S rRNA	GGCCCTGTAATTGGAATGAGT	CCAAGATCCAACTACGAGCTT
SERPINA1	CAGTGAATAAATGAGGCGTACATCC	GACTGTTTCTCATGCCTCTGGAAAG
SLCO2B1	CCTGATGCCTAGGTTTCTTTTCTTG	GGTCATCTGCCTACCCTAGAAC
ND1	TACGGGCTACTACAACCCTTC	ATGGTAGATGTGGCGGGTTT
ND5	CATTACTAACAACATTTCCCCCGC	GGCTGTGAGTTTTAGGTAGAGGG

Supplemental Table S7: Media formulation, Differentiation factors and protocol for SC-islets differentiation.

Base Media Formulation

**Reagent**	**BE1**	**BE2**	**BE3**	**S5**	**S6**	**Company**	**Catalog #**
MCDB131	500 mL	500 mL	500 mL	500 mL	500 mL	Cellgro	15-100-CV
Glucose	0.8 g	0.4 g	0.22 g	1.8 g	0.23 g	MilliporeSigma	G7528
NaHCO3	0.587 g	0.587 g	0.877 g	0.877 g	N/A	MilliporeSigma	S3817
FAF-BSA	0.5 g	0.5 g	10 g	10 g	10.5 g	Proliant	68700
ITS-X	N/A	N/A	2.5 mL	2.5 mL	N/A	Invitrogen	51500056
Glutamax	5 mL	5 mL	5 mL	5 mL	5.2 mL	Invitrogen	35050079
Vitamin C	N/A	22 mg	22 mg	22 mg	N/A	MilliporeSigma	A4544
Heparin	N/A	N/A	N/A	5 mg	10 µg/mL	MilliporeSigma	H3149
Pen/Strep	N/A	N/A	N/A	5 mL	5.2 mL	Cellgro	30-002-CI
Non-Essential Amino Acids	N/A	N/A	N/A	N/A	5.2 mL	Corning	25-025-CI
$ZnSO_4$	N/A	N/A	N/A	N/A	1 µM	MilliporeSigma	10883
Trace Elements A	N/A	N/A	N/A	N/A	523 µL	Corning	25-021-CI
Trace Elements B	N/A	N/A	N/A	N/A	523 µL	Corning	25-022-CI

Differentiation Factor Details

**Name**	**Company**	**Part Number**
Y27632	Abcam	ab120129
Chir99021	Abcam	ab120890
Alk5i II	Enzo Life Sciences	ALX-270-445-M005
ActivinA	R&D Systems	338-AC-01M
KGF	Peprotech	AF-100-19-1MG
XXI	EMD Millipore	5657901MG
L-3,30 ,5-Triiodothyronine (T3)	EMD Millipore	64245250MG
Sant1	Sigma	S4572 -5MG
Retinoic acid (RA)	Sigma	R2625-500MG
LDN193189	Sigma	SML0559 -5MG
TPPB - 1MG	R&D SYSTEMS	53431

Differentiation Protocol

	Day	Media	Factor	Final Concentration
**Stage 0**	1	mTeSR1	Y27632	10 μM
**Stage 1**	2	BE1	Activin A<br>CHIR99021	100 ng/mL<br>3 μM
	3	BE1	Activin A	100 ng/mL
	4	BE1	Activin A	100 ng/mL
	5	BE1	Activin A	100 ng/mL
**Stage 2**	6	BE2	KGF	50 ng/mL
	7	BE2	KGF	50 ng/mL
**Stage 3**	8	BE3	LDN193189<br>KGF<br>SANT1<br>RA<br>TPPB	200 nM<br>50 ng/mL<br>0.25 μM<br>2 μM<br>500 nM
	9	BE3	LDN193189<br>KGF<br>SANT1<br>RA<br>TPPB	200 nM<br>50 ng/mL<br>0.25 μM<br>2 μM<br>500 nM
**Stage 4**	10	BE3	KGF<br>SANT1<br>RA<br>TPPB<br>LDN193189	50 ng/mL<br>0.25 μM<br>0.1 μM<br>500 nM<br>200 nM
	11	BE3	KGF<br>SANT1<br>RA<br>TPPB<br>LDN193189	50 ng/mL<br>0.25 μM<br>0.1 μM<br>500 nM<br>200 nM
	12	BE3	KGF<br>SANT1<br>RA<br>TPPB<br>LDN193189	50 ng/mL<br>0.25 μM<br>0.1 μM<br>500 nM<br>200 nM
	13	BE3	KGF<br>SANT1<br>RA<br>TPPB<br>LDN193189	50 ng/mL<br>0.25 μM<br>0.1 μM<br>500 nM<br>200 nM

Differentiation Protocol (continued)

	Day	Media	Factor	Final Concentration
**Stage 5**	14	S5	SANT1<br>RA<br>XXI<br>Alk5i<br>T3<br>Latrunculin A<br>Betacellulin	0.25 μM<br>0.1 μM<br>1 μM<br>10 μM<br>1 μM<br>1 μM<br>20 ng/mL
	15	S5	SANT1<br>RA<br>XXI<br>Alk5i<br>T3<br>Betacellulin	0.25 μM<br>0.1 μM<br>1 μM<br>10 μM<br>1 μM<br>20 ng/mL
	16	S5	SANT1<br>RA<br>XXI<br>Alk5i<br>T3<br>Betacellulin	0.25 μM<br>0.1 μM<br>1 μM<br>10 μM<br>1 μM<br>20 ng/mL
	17	S5	SANT1<br>RA<br>XXI<br>Alk5i<br>T3<br>Betacellulin	0.25 μM<br>0.1 μM<br>1 μM<br>10 μM<br>1 μM<br>20 ng/mL
	18	S5	SANT1<br>RA<br>XXI<br>Alk5i<br>T3<br>Betacellulin	0.25 μM<br>0.1 μM<br>1 μM<br>10 μM<br>1 μM<br>20 ng/mL
	19	S5	SANT1<br>RA<br>XXI<br>Alk5i<br>T3<br>Betacellulin	0.25 μM<br>0.1 μM<br>1 μM<br>10 μM<br>1 μM<br>20 ng/mL

Differentiation Protocol (continued)

	Day	Media	Factor	Final Concentration
**Stage 5**	20	S5	SANT1<br>RA<br>XXI<br>Alk5i<br>T3<br>Betacellulin	0.25 μM<br>0.1 μM<br>1 μM<br>10 μM<br>1 μM<br>20 ng/mL
**Stage 6**	Feed S6 every other day			

Supplemental Table S8: Hashtag antibody list used in this study.

Antibody (Biolegend)	Sequence	Catalog #
TotalSeq™-A0251 anti-human Hashtag 1 Antibody	GTCAACTCTTTAGCG	394601
TotalSeq™-A0252 anti-human Hashtag 2 Antibody	TGATGGCCTATTGGG	394603
TotalSeq™-A0253 anti-human Hashtag 3 Antibody	TTCCGCCTCTCTTTG	394605
TotalSeq™-A0254 anti-human Hashtag 4 Antibody	AGTAAGTTCAGCGTA	394607
TotalSeq™-A0255 anti-human Hashtag 5 Antibody	AAGTATCGTTTCGCA	394609
TotalSeq™-A0256 anti-human Hashtag 6 Antibody	GGTTGCCAGATGTCA	394611
TotalSeq™-A0257 anti-human Hashtag 7 Antibody	TGTCTTTCCTGCCAG	394613
TotalSeq™-A0258 anti-human Hashtag 8 Antibody	CTCCTCTGCAATTAC	394615
